# Supplementary material for: Added value of assessing medical students’ reflective writings in communication skills training: a longitudinal study in four academic centres
Source: BMJ Open. 2020 Nov 6;10(11):e038898. doi: 10.1136/bmjopen-2020-038898 (PMC7651724; doi:10.1136/bmjopen-2020-038898)
Supplement: Supplementary data [file bmjopen-2020-038898supp001.pdf]

Appendix 1 - Scoring Reflective Writing using REFLECT rubric:

**Reflective Writing:** *I felt uncomfortable and it was hard for me stay present in the consultation because of the way the professor informed the diagnosis and managed the patient. Assessing the situation according to what physicians must do, several skills were not fulfilled in the patient care process: attention to patient well-being, autonomy and responsibility to promote better health for patients. The gathering of history by students had no benefit to the patient and only served a didactic function. As the diagnosis is cancer, which is stigmatised and has a very high negative charge (senior physician had performed a prior consultation and obtained all necessary information), it might not be the best time for medical students "to practice" history-taking. After our history-taking, the senior physician discussed the therapy for cancer with students and asked another physician to participate. They discussed the prognosis for the patient, suggested a new protocol in the research phase and assumed results that should not happen. All of these events occurred in front of the patient and their family. Adequate communication is important to adapt communication to each patient. Information must be provided according to subjects' needs and their capacity to understand… "Why to discuss in that way? They discussed uncertain things and affirmed the prognosis and other indications without scientific confirmation. It is difficult to evaluate these complex issues as students due to the scarce theoretical foundation for communication in medical school. The process of assimilation and application of role models prevails if there is no other point of criticism…*

**1- Writing Spectrum – Level: Reflection** ("movement beyond reporting or descriptive writing to reflecting; i.e., attempting to understand, question, or analyse an event"[1]). The fragments disposed of reveal that students wrote beyond the descriptive level. However, they did not explore and criticise the values, believes or assumptions behind the observed behaviour. Thus, this reflection exceeds the descriptive level and achieves reflection but not a critical reflection – the higher level for writing spectrum: *"The gathering of history by students had no benefit for the patient, but only a didactic function."; "As the diagnosis is cancer, which is stigmatised and has a very high negative charge (senior physician had conducted a prior consultation and obtained all necessary information), it might not be the best time for medical students "to practice" history-taking."*

**2- Presence – Level: Reflection** ("sense of writer being largely present"[1]) – The students presented the situation including her/himself in the situation, described the situation according to her/his point of view, which enabled an understanding of the participation of the student in the consultation. However, more details are needed to bring the reader to the setting, as expected for the Critical Reflection Level.

**3- Description of conflict or disorienting dilemma – Level: Reflection** ("description of the disorienting dilemma, conflict, challenge, or issue of concern"[1]) – The description includes the disorienting dilemma but does not include a more profound understanding of the "conflict, challenge, or issue of concern that includes multiple perspectives…" as expected for the next level: "Critical Reflection". There are three main dilemmas: the need to adapt the communication to each patient, the negative role models and the responsibility to patient well-being. All these elements were clearly stated in the text but lacked the necessary detail for Critical Reflection.

**4- Attending to Emotions – Level: Thoughtful action** ("recognition but no exploration or attention to emotions"[1]) – The students described his/her feeling and the narrative transmits his/her difficulty in handling emotions during the situation. However, no exploration was required for the next level of writing (Reflection) and beyond the recognition and insight on emotions necessary in Critical Reflection.

**5- Analysis and Meaning Making – Level: Reflection** ("some analysis and meaning-making"[1]) - The student noticed problems regarding communication and physicians' attitude. The writing suggests that the students recognised and analysed the situation; however, it could be more comprehensive for achieving Critical Reflection – for example, why did this doctor behave in this manner? The following fragments present some analysis of the student: *"it might not be the best time for medical students "to practice" history-taking..."; "To communicate adequately is important to adapt communication to each patient, and the information must be provided according to subjects' needs and the capacity to understand…".*

1- REFLECT rubric statements from: Wald, H. S., Borkan, J. M., Taylor, J. S., Anthony, D., & Reis, S. P. (2012). Fostering and Evaluating Reflective Capacity in Medical Education: Developing the REFLECT Rubric for Assessing Reflective Writing. Academic Medicine, 87(1), 41–50. The text in Italic correspond to the student reflective writing. The text in bold correspond to the REFLECT rubric items.
