# Supplementary material for: Added value of assessing medical students’ reflective writings in communication skills training: a longitudinal study in four academic centres
Source: BMJ Open. 2020 Nov 6;10(11):e038898. doi: 10.1136/bmjopen-2020-038898 (PMC7651724; doi:10.1136/bmjopen-2020-038898)
Supplement: Author's manuscript [file bmjopen-2020-038898.draft_revisions.pdf]

BMJ Open is committed to open peer review. As part of this commitment we make the peer review history of every article we publish publicly available.

When an article is published we post the peer reviewers' comments and the authors' responses online. We also post the versions of the paper that were used during peer review. These are the versions that the peer review comments apply to.

The versions of the paper that follow are the versions that were submitted during the peer review process. They are not the versions of record or the final published versions. They should not be cited or distributed as the published version of this manuscript.

BMJ Open is an open access journal and the full, final, typeset and author-corrected version of record of the manuscript is available on our site with no access controls, subscription charges or pay-per-view fees (http://bmjopen.bmj.com).

If you have any questions on BMJ Open's open peer review process please email info.bmjopen@bmj.com

BMJ Open

The added value of assessing medical students' reflective writings in communication skills training: a longitudinal study in four academic centres.

Journal:	*BMJ Open*
Manuscript ID	bmjopen-2020-038898
Article Type:	Original research
Date Submitted by the Author:	31-Mar-2020

Complete List of Authors:	Ament Giuliani dos Santos Franco, Camila; Pontifical Catholic University of Parana, Medicine School<br>Franco, Renato; Pontifical Catholic University of Parana, Medicine School<br>Cecilio-Fernandes, Dario; State University of Campinas Faculty of Medical Sciences, ; University Of Groningen, University Medical Center Groningen<br>Severo, Milton; University of Porto Medical School, Department of Clinical Epidemiology, Predictive Medicine and Public Health and Public Health and Forensic Sciences, and Medical Education Department<br>Ferreira, Maria; University of Porto Faculty of Medicine, Public Health and Forensic Sciences, and Medical Education Department<br>de Carvalho-Filho, Marco Antonio; University Medical Center Groningen, CEDAR - Center for Educational Development and Research in Health Sciences; Universidade Estadual de Campinas Faculdade de Ciencias Medicas, Internal Medicine

Keywords:	MEDICAL EDUCATION & TRAINING, MEDICAL ETHICS, PRIMARY CARE

SCHOLARONE™
Manuscripts

I, the Submitting Author has the right to grant and does grant on behalf of all authors of the Work (as defined in the below author licence), an exclusive licence and/or a non-exclusive licence for contributions from authors who are: i) UK Crown employees; ii) where BMJ has agreed a CC-BY licence shall apply, and/or iii) in accordance with the terms applicable for US Federal Government officers or employees acting as part of their official duties; on a worldwide, perpetual, irrevocable, royalty-free basis to BMJ Publishing Group Ltd ("BMJ") its licensees and where the relevant Journal is co-owned by BMJ to the co-owners of the Journal, to publish the Work in this journal and any other BMJ products and to exploit all rights, as set out in our licence.

The Submitting Author accepts and understands that any supply made under these terms is made by BMJ to the Submitting Author unless you are acting as an employee on behalf of your employer or a postgraduate student of an affiliated institution which is paying any applicable article publishing charge ("APC") for Open Access articles. Where the Submitting Author wishes to make the Work available on an Open Access basis (and intends to pay the relevant APC), the terms of reuse of such Open Access shall be governed by a Creative Commons licence – details of these licences and which Creative Commons licence will apply to this Work are set out in our licence referred to above.

Other than as permitted in any relevant BMJ Author's Self Archiving Policies, I confirm this Work has not been accepted for publication elsewhere, is not being considered for publication elsewhere and does not duplicate material already published. I confirm all authors consent to publication of this Work and authorise the granting of this licence.

For peer review only

The added value of assessing medical students' reflective writings in communication skills training: a longitudinal study in four academic centres.

Assessing reflective writings in communication skills training

Camila Ament Giuliani Franco

Medicine School, Pontifical Catholic University of Paraná, Curitiba, Brazil.

Address: Rua Imaculada Conceição, 1155 - Prado Velho, Curitiba – PR, 80215-901, Brasil - Escola de Medicina – PUCPR (Medicine School). Tel. +55 41 3271-1570. Email: camilaament@gmail.com; camila.giuliani@pucpr.br ORCID: https://orcid.org/0000-0003-1176-480X

Family Physician, MD, PhD. Professor at the School of Medicine (discipline of Family Medicine), Pontifical University of Paraná, Curitiba, Brazil. Public Health and Forensic Sciences, and Medical Education Department, University of Porto, Portugal.

The author declared no potential conflicts of interest with respect to the research, authorship, and/or publication of this article

Renato Soleiman Franco

Medicine School, Pontifical Catholic University of Paraná, Curitiba, Brazil.

Address: Rua Imaculada Conceição, 1155 - Prado Velho, Curitiba – PR, 80215-901, Brasil - Escola de Medicina – PUCPR (Medicine School). Tel. +55 41 3271-1570. Email: paum@uol.com.br; renato.soleiman@pucpr.br. ORCID: https://orcid.org/0000-0003-1176-480X

Psychiatrist, MD, PhD. Professor at the School of Medicine (discipline of Psychiatry), Pontifical University of Paraná, Curitiba, Brazil. Public Health and Forensic Sciences, and Medical Education Department, University of Porto, Portugal. Director of the Psychiatry Residency Program – FEAES/PMC, Curitiba.

For peer review only - http://bmjopen.bmj.com/site/about/guidelines.xhtml

The author declared no potential conflicts of interest with respect to the research, authorship, and/or publication of this article

Dario Cecilio-Fernandes

Department of Medical Psychology and Psychiatry, School of Medical Sciences, University of Campinas (UNICAMP), Campinas, SP, Brazil.

Address: R. Tessália Vieira de Camargo, 126 - Cidade Universitária, Campinas - SP, 13083-887. dario.fernandes@gmail.com. ORCID: https://orcid.org/0000-0002-8746-1680

Psychologist. PhD - Researcher at School of Medical Sciences, University of Campinas (UNICAMP), Campinas, SP, Brazil and Researcher, AFAMEE.

The author declared no potential conflicts of interest with respect to the research, authorship, and/or publication of this article

Milton Severo

Public Health and Forensic Sciences, and Medical Education Department. Faculty of Medicine, University of Porto, Portugal.

Address: Milton Severo, Departamento de Ciências da Saúde Pública e Forenses e Educação Médica, Piso 6, Faculdade de Medicina da Universidade do Porto, Alameda Prof. Hernâni Monteiro, 4200-319 Porto – Portugal. E-mail: severo.milton@gmail.com; milton@med.up.pt ORCID: https://orcid.org/0000-0002-5787-4871.

Graduated in Applied Mathematics and Computation, PhD in Public Health. Department of Epidemiology, Predictive Medicine and Public Health, Faculty of Medicine, University of Porto, Portugal. Public Health and Forensic Sciences, and Medical Education Department, University of Porto, Portugal. EPIUnit - Institute of Public Health, University of Porto, Portugal.

For peer review only - http://bmjopen.bmj.com/site/about/guidelines.xhtml

The author declared no potential conflicts of interest with respect to the research, authorship, and/or publication of this article.

Maria Amélia Ferreira

Public Health and Forensic Sciences, and Medical Education Department. Faculty of Medicine, University of Porto, Portugal.

Address: Departamento de Ciências da Saúde Pública e Forenses e Educação Médica, Piso 6, Faculdade de Medicina da Universidade do Porto, Alameda Prof. Hernâni Monteiro, 4200-319 Porto – Portugal. Email: mameliaferreira@gmail.com; mameliaferreira@med.up.pt. ORCID: https://orcid.org/0000-0001-6789-3796.

Physician, PhD in Medical Education. Public Health and Forensic Sciences, and Medical Education Department, University of Porto, Portugal.

The author declared no potential conflicts of interest with respect to the research, authorship, and/or publication of this article

Marco Antonio de Carvalho Filho (corresponding author)

University of Campinas, Campinas, SP, Brazil and Center for Education Development and Research in Health Professions - Research Group LEARN - Lifelong Learning, Education & Assessment Research, University of Groningen, Groningen, The Netherlands.

Address: Antonius Deusinglaan, 4, 9713 AW, Groningen, The Netherlands. E-mail: m.a.de.carvalho.filho@umcg.nl. https://orcid.org/0000-0001-7008-4092

Emergency Physician. PhD, Associate Professor, Internal Medicine Department, School of Medical Sciences, University of Campinas, Brazil; and Senior Researcher, Center of Education Development and Research in Health Professions, Research Group LEARN, University Medical Center Groningen, The Netherlands.

For peer review only

The added value of assessing medical students' reflective writings in communication skills training: a longitudinal study in four academic centres.

Abstract

Objectives: This study describes the development and implementation of a model to assess students` communication skills highlighting the use of reflective writing. We aimed to evaluate the usefulness of the students' reflections in the assessment of communication skills.

Design: Third- and fourth-year medical students enrolled in an elective course on clinical communication skills development were assessed using different assessment methods.

Setting and Participants: The communication skills course was offered at four universities (three in Brazil and one in Portugal) and included 69 students.

Outcome measures: The students were assessed by a multiple-choice questionnaire (MCQ), an objective structured clinical examination (OSCE), and reflective writing narratives. The Cronbach's alpha, Dimensionality, and the Person's correlation were applied to evaluate the reliability of the assessment methods and their correlations. The depth of reflection was evaluated using the REFLECT Rubric (Reflect Score [RS]), and the themes of reflection were assessed by a Thematic Score (TS).

Results: The MCQ Cronbach's alpha was 0.697; for the six OSCE stations (OSCE global score), the Cronbach's alpha was 0.633. The RS had a Cronbach's alpha of 0.850 with an inter-examiner correlation of 0.816. In the TS, the Cronbach's alpha was 0.784, and the inter-examiner correlation was 0.907. The assessment of reflection using the TS was significantly correlated MCQ ($r=0.412$; $p=0.019$), OSCE (0.439; p=0.012*), and RS (0.410; $p=0.020$). However, the depth of reflection (RS) only correlated with the TS.

Conclusions: The use of reflective writing expands the assessment of communication skills. Assessing reflection implies not only identifying the themes of the reflection but also its depth. Indeed, reflective depth seems to be a specific competence, not correlated with other assessment methods - possibly a metacognitive domain.

Keywords: medical education & training; medical ethics; primary care.

For peer review only - http://bmjopen.bmj.com/site/about/guidelines.xhtml

Strengths and limitations of this study

- This study details the use of medical students' reflective narratives in the assessment of communication skills.
- The assessment of the depth (profundity) and the themes (topics) of medical students' reflective narratives has an additional value compared to the traditional assmesment methods used in communication skills training.
- The method utilized to assess the depth and themes of medical students' reflective narratives showed good reliability.
- The participants were recruited from a convenience sample and further studies are needed to explore the added value of assessing medical students' reflective naratives in a natural context.

For peer review only

1. Introduction

Clinical communication is essential for medical students and must extend well beyond the reproduction of behaviours and skills [1]. Competent doctors must adapt their communication to the specific needs of their patients [2]. In this regard, for medical students to become competent communicators, they must reflect on their experiences with patients aiming for the self-monitoring of their thoughts and behaviours [1,3]. This reflective habitus may also help students to handle the particularities of each one of their clinical encounters, adapting their communication style to patients` needs and preferences [4]. Thus, reflection is an essential component of communication. However, most communication skills training does not include the assessment of students` reflections in their repertoire of assessment tools [5]. Understanding how assessing reflection supports (or not) the development of communication skills in medical students may offer medical educators a new strategy for improving doctor-patient communication.

The learning of communication skills requires that medical students adapt and adjust their patterns of behaviour to the needs and context of their patients [6]. Although the learning of some basic behavioural rules can indeed be an excellent starting point, such rules governing behaviour may not suffice for guiding students in the process of navigating the

For peer review only - http://bmjopen.bmj.com/site/about/guidelines.xhtml

complexity of doctor-patient communication. Each patient is unique and has his or her system of beliefs and singular expectations. Doctors must reflect in and on action to tailor their communication strategies to match each patient needs while respecting his or her personality and social and cultural background [7,8]. Therefore, we believe that the educational activities of the medical curricula targeting communication skills development should also include the teaching and assessment of reflection.

Within an educational context, reflection is a process [9] whereby an individual critically analyses a previous experience and develops a deeper understanding of that experience. This 'reflection-on-action' plays a vital role in building mental models to be applied in clinical contexts. During the reflection, the subject must have self-awareness and engage in self-monitoring (metacognition) to guide his or her thoughts about a particular situation eliciting in them the disposal to reflect [3]. Although reflection has been considered keen in the development of clinical communication, its implementation has a low degree of systematisation, and little attention has been paid to the descriptions of the use of reflection as an assessment tool.

Reflection can be assessed based on its content or depth. The content of reflection may be evaluated by theme or category-based analysis [10–12]. For example, Karnieli-Miller et al. (2018) used reflective writing to support the teaching of how to deliver bad news. In the reflective narratives, the authors identified through theme-based analysis all the elements that were part of the clinical protocol used as a reference during the study [11]. However, the study focused only on the content of reflection, but not on the depth of reflection. Moreover, the authors did not compare the results of the assessment of the reflection with those obtained through other methods of assessment. Similar to Karnieli-Miller et al. (2018), Braverman et al. (2016) used a coded framework for the thematic analysis of third-year medical students' reflective writing on challenges in communicating with patients but also did not assess the depth of reflection [12]. Thus, the studies that have sought to determine the role of reflection in teaching communication have targeted its themes, rather than its depth.

The Reflection Evaluation for Enhanced Competencies Tool (REFLECT rubric), proposed by Wald et al. (2012), highlights the importance of deep reflection in the development of metacognition and effective patient care [13] and has been widely used to evaluate reflection [14]. These authors organised a multidimensional analysis of reflection that assesses five mandatory items: description, presence, identification of a dilemma,

For peer review only - http://bmjopen.bmj.com/site/about/guidelines.xhtml

emotion, and the meaning of the experience [13]. These five items can be classified using a Likert scale ranging from 0 to 3, according to four different reflection levels (from non-reflective to critically reflective), which correspond to the depth of reflection. This assessment model distinguishes between written texts with superficial reflection (descriptive) and those with a high density of reflective elements. Although the REFLECT rubric was used successfully in assessment strategies for different learning activities in the medical education domain, the authors did not find any report of its use in communication skills training.

Communication training traditionally applies a combination of multiple choice questionnaires (MCQs) and Objective Structured Clinical Examination (OSCE) stations to assess students' cognitive knowledge and check students' performance [15,16]. Research shows a low correlation between the MCQ and OSCE scores, which suggests that, indeed, these methods are assessing different competencies [17–19]. Communication teachers can take advantage of these different scores and provide specific feedback targeting knowledge and/or behaviour. If cultivating reflection skills is also relevant to the process of becoming a good communicator, communication trainers should implement assessment strategies that target reflection skills to create an opportunity to provide feedback on this competency [20]. Moreover, if reflection is a specific domain of competency, as we believe, the scores for reflection will have a low level of correlation with scores for traditional assessments such as MCQs and OSCEs. Therefore, understanding how the assessment of the content and depth of students' reflection add (or not) to conventional assessment methods can enlighten the importance of reflection in the process of honing communication skills to improve doctor-patient communication.

In this study, we report the development of a model for assessing the reflection process of medical students in the context of communication skills training. We also compare the assessment of reflection with other traditional methods (i.e. MCQ and OSCE) to understand the added value of assessing reflection. Our assumptions are: (1) reflection themes will correlate to MCQ scores, as both are related to knowledge, and (2) MCQ, OSCE, and reflection depth will not show correlations, as they measure different competencies (knowledge, skills, and reflective thinking, respectively).

2. Methods

For peer review only - http://bmjopen.bmj.com/site/about/guidelines.xhtml

2.1 Overview

This longitudinal observational study was carried out at three different Brazilian universities (one course at each university in 2015) and one university in Portugal (one course in 2016). Data collection occurred during these elective courses in clinical communication. Each course comprised five modules (25 hours in total) conducted over two months. The Calgary-Cambridge Guide to Communication [21,22] and Patient-Centred Medicine [23] were the conceptual and theoretical models behind the course. Those models were used as supportive frameworks, and students were not encouraged to follow them as behavioural protocols. The main focus of the course was on the need to reflect and adapt communication strategies to patients' needs and students' communication style. The modules of the course presented the content through reflective, small-group discussions, followed by simulation activities that used simulated patients and debriefing [24]. A detailed discussion of the course has been previously published [24].

2.2 Participants

A convenience sample of third- and fourth-year medical students at four universities were invited to participate in the study by email. For the sample recruitment, a class representative of the students in the third or fourth year sent an email to their colleagues inviting them to participate in the course. No financial incentives were given for their participation. A total of 69 participants (20 at University 1 - Brazil, 12 at University 2 - Brazil, 30 at University 3 - Brazil, and seven at University 4 - Portugal) agreed to participate. The participants joined a course containing five encounters with a total of 25 hours. The 69 participants were assessed at the end of the course with an MCQ and OSCE on communication skills. The participants were invited (but not obligated) to write a reflective piece, and 37 students produced texts.

2.3 Material: Assessment Instruments

We compared three different assessment methods: a cognitive test based on an MCQ, an examination of communication skills based on the OSCE, and an assessment of reflection

For peer review only - http://bmjopen.bmj.com/site/about/guidelines.xhtml

through reflective writing. The MCQ and OSCE were administered after the last meeting of the course. The reflective writing was optional and could be undertaken by the students at any point during the course. We decided that the reflective writing would be optional to understand the students' disposition to engage with this assessment method [25].

The MCQ consisted of 63 items about clinical communication and the OSCE included six stations (specifically designed to assess communication skills); four of these had been tested in a pilot project [26]. This assessments were developed together with the Medical Education Department of the University of Porto to guarantee the quality of the items and stations. The MCQs and the checklist items were based on clinical situations or conceptual issues related to reference frameworks as the Calgary-Cambridge Guide to Communication [21,22], Patient-Centred Medicine [23] and the Kalamazoo Consensus [27].

There was one observer for each OSCE station who was responsible for filling out the assessment checklist. These checklists consisted of between six and 14 items per station. Each item on the checklist was then classified on a Likert scale ranging from 0 to 2 points. The final score of each station was obtained by the mean of its items. The OSCE global score was calculated as a mean considering the six stations.

For the reflective writing component, students could choose any aspect of communication to explore, following the instruction: 'Suggestion for reflection: 1) Describe the situation; 2) Point out the dilemmas, doubts, and questions raised; 3) Point out feelings and observations; 4) Analyse the situation from different points of view; 5) Make a conclusion; and 6) Suggest a hypothesis. These steps are only a suggestion; you may conduct the reflection in whichever way that you prefer'. The reflective writing was evaluated in two different ways: (1) through the sum of the themes covered in each one of reflections – the thematic score (TS), and (2) through the REFLECT Rubric – the reflect score (RS) [13]. In the next paragraphs we describe how these two scores were calculated.

For establishing the TS, two researchers (CF and RF) started a content analysis individually by reading carefully all the reflective writings made by the students. After reading, CF and RF selected the fragments related to clinical communication [28] and generated a single list with all the fragments from the reflections of all students. Next, CF and RF grouped the fragments in thematic categories independently. After, CF and RF met to reach a consensus on the main themes. After the definition of the main thematic categories, CF and RF read each one of the reflective writings for a second time and decided whether

For peer review only - http://bmjopen.bmj.com/site/about/guidelines.xhtml

each of the themes were present or not. The two researchers assigned point scores accordingly to the presence of a certain theme ('0' for absent and '1' for present). The final TS corresponded to the sum of all the themes approached by the student. Finally, the agreement between the two researchers was evaluated, and, when there was a difference between the two, a final TS was reached by consensus.

The assessment based on the five mandatory dimensions of the REFLECT Rubric followed the guidelines set by the authors of the rubric. The five mandatory dimensions are: description, presence, identification of a dilemma, emotion, and the meaning of the experience. Each one of the dimensions are evaluated considering four levels of reflective capacity scored from 0-3 (habitual action or nonreflective = 0, thoughtful action or introspection = 1, reflection = 2 and critical reflection = 3). The sum of the scores obtained in each dimension was the total RS. (Table 1)

[Table 1 near here]

In summary, the TS refers to 'the subject of reflection – number of themes', the RS refers to 'how the reflection took place or the depth of reflection'.

2.4 Analysis

To assess the quality of the quantitative instruments, MCQ and OSCE, we performed an analysis of the main components and consistency based on Cronbach's alpha. A principal components analysis was used to assess dimensionality and content validity. Dimensionality was assessed using a scree plot, and the number of components was assessed according to the 'elbow rule'. An element or item was considered to contribute to a principal component when it had a correlation value higher than 0.30. Internal consistency was evaluated using Cronbach's alpha (Cronbach 1951). Acceptable values for internal consistency were considered to be higher than 0.7. The linear associations between the assessment methods were assessed using the Pearson correlation considering missing complete at random to handle with missed correlations.

To measure agreement between researchers, we used the intraclass single average value for absolute agreement. The inter-rater agreement rate was calculated for encoded

For peer review only - http://bmjopen.bmj.com/site/about/guidelines.xhtml

fragments (TS) and for the RS. NVivo software (version 11.3.2 for Mac) was used for qualitative data analysis, while the SPSS, Version 25.0, was used for quantitative data analysis.

This research was approved by the Ethics Centre of the São João Hospital Centre of the Faculty of Medicine of the University of Porto (FMUP) and by the Research and Ethics Commission of the Pontifical Catholic University of Paraná (PUCPR). Participant consent was requested in the form of an informed consent before the participation in the communication skills course. Signed written consent forms were completed by all participants.

2.5 Patient and Public Involvement

There is no patient involved in the study

3. Results

Sixty-nine students followed the courses and were included in the study. Fifty-five of the students were women (79.7%), and the mean age of participants was 23.5 years (SD 2.495). Fourth-year students were the largest cohort (69.6%). All participants (69 students) underwent the MCQ and OSCE examinations, and 32 students also performed the reflective writing.

3.1 Quality of the Instruments

The MCQ examination had a Cronbach's alpha of 0.697. For the six OSCE stations, the lower Cronbach's alpha level was 0.702, and the higher was 0.815. The Cronbach's alpha of the OSCE global score was 0.633.

The TS had a Cronbach's alpha of 0.784, while the inter-examiner correlation for absolute single-measure concordance was 0.907 (two examiners). The RS had a Cronbach's alpha of 0.850 and an inter-examiner correlation for absolute single-measure concordance of 0.816 (two examiners).

For peer review only - http://bmjopen.bmj.com/site/about/guidelines.xhtml

3.2 Thematic Analysis

The thematic categories of the reflections were non-verbal communication (NV), the patient's perspective (PP), steps of communication (SC), doctor-patient relationships (DPR), ethics and respect (ER), empathy and altruism (EA) and humanistic values (HV) (Table 2).

[Table 2 near here]

3.3 Correlation between Instruments

Table 3 shows the correlations between the four different assessment methodologies. There was no correlation between the score for the depth of reflection (RS) and both the MCQ and OSCE scores. The RS was only correlated with the thematic score (TS). However, the TS score was positively correlated with the MCQ score (0.439; p=0.012) and the OSCE score (0.412; p=0.019).

[Table 3 near here]

4. Discussion

The assessment of the depth and themes of reflection provides a different perspective on the teaching and learning of communication skills. We found a positive correlation between the content of the students' reflections with their performance on a cognitive test and OSCE assessment, which suggested that the scope of the reflection was related to the students' knowledge. The lack of correlation between the depth of reflection and cognitive and behavioural tests suggests that reflection could be a particular competence domain.

For peer review only

4.1 Importance of Including Assessment of Depth and Content When Evaluating Reflection

The reflection process ranges from elementary cognitive levels (description, identification, knowledge, and others) to higher levels of processing, such as analysis, evaluation, synthesis, and creation [29]. Using different methods to assess reflection offers an effective strategy to encourage students to engage in reflective activities and enhances the probability of students reaching deeper levels of reflection [30]. Thus, we agree with Hulsman et al. (2009) and advocate for the assessment of reflection in terms of its depth and content to improve communication skills training [31].

In the assessment of the reflection themes, teachers map the topics students address in their reflections. We observed that the number of themes addressed by students are linked to both knowledge [29] and practical performance [32–34]. We believe that a broader knowledge helps students to respond appropriately to different practical situations.

Interestingly, the reflection depth seems to be a different competence, not necessarily related to the knowledge level or current performance, but possibly related to the values and attitudes of the student regarding a specific topic [31]. Aligned with this hypothesis, Moniz et al. (2015) showed a lack of correlation between the depth of reflection (RS) and OSCE and MCQ scores of undergraduate medical students. However, in Moniz`s study, the assessment methods were not targeting the same competence and the absence of standardization could explain the observed lack of correlation. In our study, we assessed a singular set of competencies (communication skills) and observed the same lack of correlation between reflection depth and other assessment methods.

Learning is a lifelong enterprise and achieving deeper reflection is crucial to the process of becoming an independent and self-regulated learner [35].The achievement of deeper reflection requires (1) understanding the context; (2) elaborating on the experience; (3) searching for solutions to the problems posed; (4) acknowledging the different subjects involved; and (5) taking different perspectives [36]. Thus, when doctors achieve a deep sense of reflection on their practice they move from a state of being knowledge consumers to become active professionals capable of transforming their reality aiming for a practice based on their values and centred on the patient [37]. We believe that deeper reflection goes beyond applying the knowledge to a fixed situation; deeper reflection incorporates the elaboration of

For peer review only - http://bmjopen.bmj.com/site/about/guidelines.xhtml

new knowledge, balance of different perspectives, anticipation of challenges and planning of future behaviour[39].

Adding the depth of reflection to teaching and assessment models may allow teachers to capture students' standpoint, their meaning-making processes, and their values [40]. We hypothesize that the depth of reflection, particularly concerning communication skills, could be linked to the domain of 'being a doctor' and the formation of professional identity [41,42] by involving elements that extend beyond the context of daily practice to include belief systems and values, which are not commonly assessed in knowledge tests and OSCEs.

4.2 The Risks of Assessing Reflection

The assessment of reflection brings the risk of reducing the reflective practice to a tool or a mere parameter of a grading system. Under the pressure of scoring to pass exams, students often "play the game" and perform tasks and adopt behaviours to fit the expectations of teachers, without making a real transformative engagement in learning [43]. This risk should be avoided by linking the reflective process to the questioning of assumptions, power relations, and social and systemic structures. Ideally, the assessment of reflection should go in the opposite direction of conventional assessment methods [44]. The lack of correlation observed between depth of reflection and MCQ or OSCE scores aligns with this notion. Nevertheless, it is essential to continue studying the role of reflection among assessment methods to develop the potential of reflective practice without being reductive [45].

4.3 Limitations

The sample of this study was small and convenient. Thus, it is difficult to generalise our results to larger samples. As it was self-selected, the sample may represent more knowledgeable and motivated students, which may influence both the scores and percentage of students who engaged in the reflective writing (higher than 50%). The fact that the reflection was optional could have attracted students who were naturally reflective, which can also be a confounder. Our results must be confirmed by investigations using non-convenient samples and with a greater number of participants.

The assessments were reliable and consistent but limited in terms of reproducibility owing to the number of assessments made. Our method of assessing reflection (reflective

For peer review only - http://bmjopen.bmj.com/site/about/guidelines.xhtml

For peer review only

writing) could be an element of bias since studies show different results when different reflection methods are used. For example, when reflecting in interviews, students may show levels of reflection that are different from those shown in reflective writing [46]. The current generation of students has a range of preferences when it comes to learning and methods of expression, and many do not have strong skills in written expression [47]. Thus, reflective depth can indeed be associated with students' writing skills [48]. In this way, some authors suggest diversification of reflective registers using alternatives such as digital storytelling. Thus, the use of writing to assume the depth of reflection has an important bias to be considered. Drawing definite conclusions about students' reflectiveness from only one source of reflective material may be biased.

4.5 Practical Implications

Becoming a good communicator is one of the challenges posed to medical students. Communication training already embraces a body of cognitive knowledge that grounds learning activities. Communication training has also developed different strategies to nurture, check, and give feedback on the behaviours and attitudes of medical students during role-playing and simulated or real clinical encounters. However, becoming a good communicator is a life-long process, and, after leaving medical school, junior doctors have to take control of their learning process. Developing a reflective mindset that is capable of evaluating current behaviour – its roots, professional and personal consequences, and emotional impact – will allow junior doctors to transform their understandings and attitudes towards more patient-centred care. Reflection can facilitate this trajectory by supporting medical students during their first steps in becoming autonomous critical thinkers.

Conclusion

This study supports the use of reflective narratives as a complementary assessment method in the context of communication skills training. Assessing the depth of reflection offers a new perspective on students' development and allows the teacher to dive into students' understandings of the value of becoming a good communicator.

For peer review only

Acknowledgements

We would also like to thank students for their willingness to participate in the course and achieve the assessment methods.

Declaration of interest

The authors report no declarations of interest.

Author Statement

Franco, CAGS and Franco, RS participated in the conception, design of the study, acquisition of data, analysis and interpretation of data, writing the final version of the manuscript. Cecilio-Fernades, D made substantial contributions revising the paper critically for important improvement in whole manuscript. Carvalho-Filho MA made substantial contributions in the analysis, interpretation of data and in the writing of the final version of the manuscript. Severo, M and Ferreira, MA made substantial contributions in the conception and design of the study, analysis and interpretation of data. All authors read and approved the final manuscript.

Declaration of Conflicting Interests

The author(s) declared no potential conflicts of interest with respect to the research, authorship, and/or publication of this article.

Funding

Financial support for the authors was provided by scholarships from the Conselho Nacional de Desenvolvimento Científico e Tecnológico (Brazilian National Council of Technological and Scientific Development - 229753/2013-2) and the Coordenação de Aperfeiçoamento de Pessoal de Nível Superior (Coordination for the Improvement of Higher Education Personnel, Brazil -13271/13-0).

For peer review only - http://bmjopen.bmj.com/site/about/guidelines.xhtml

Data availability statement: The datasets used during are available from the corresponding author on reasonable request.

Word Count: 3622 words

References

1 Stewart M, Brown JB, Donner A, *et al.* The impact of patient-centered care on outcomes. *J Fam Pract* 2000;**49**:796–804.

2 Hoffmann TC, Bennett S, Tomsett C, *et al.* Brief training of student clinicians in shared decision making: a single-blind randomized controlled trial. *J Gen Intern Med* 2014;**29**:844–9. doi:10.1007/s11606-014-2765-5

3 Sandars J. The use of reflection in medical education: AMEE Guide No. 44. *Med Teach* 2009;**31**:685–95. doi:10.1080/01421590903050374

4 General Medical Council (GMC). Outcomes for graduates 2018. Gen Med Counc. 2018;:26. doi:http://dx.doi.org/10.1016/j.healun.2004.01.011

5 Carney PA, Palmer RT, Fuqua Miller M, *et al.* Tools to Assess Behavioral and Social Science Competencies in Medical Education. *Acad Med* 2016;**91**:730–42. doi:10.1097/ACM.0000000000001090

6 Deveugele M. Communication training: Skills and beyond. *Patient Educ Couns* 2015;**98**:1287–91. doi:10.1016/j.pec.2015.08.011

7 Sommer J, Lanier C, Perron NJ, *et al.* A teaching skills assessment tool inspired by the Calgary-Cambridge model and the patient-centered approach. *Patient Educ Couns* 2016;**99**:600–9. doi:10.1016/j.pec.2015.11.024

8 Mamede S, Schmidt HG. The structure of reflective practice in medicine. *Med Educ* 2004;**38**:1302–8. doi:10.1111/j.1365-2929.2004.01917.x

9 Nguyen QD, Fernandez N, Karsenti T, *et al.* What is reflection? A conceptual analysis of major definitions and a proposal of a five-component model. *Med Educ* 2014;**48**:1176–89. doi:10.1111/medu.12583

For peer review only - http://bmjopen.bmj.com/site/about/guidelines.xhtml

10 Karnieli-Miller O, Palombo M, Meitar D. See, reflect, learn more: qualitative analysis of breaking bad news reflective narratives. *Med Educ* 2018;**52**:497–512.

11 Karnieli-Miller O, Michael K, Eidelman S, *et al.* What you "see" is how you communicate: Medical students' meaning making of a patient's vignette. *Patient Educ Couns* 2018;**101**:1645–53. doi:10.1016/j.pec.2018.04.004

12 Braverman G, Bereknyei Merrell S, Bruce JS, *et al.* Finding the Words: Medical Students' Reflections on Communication Challenges in Clinic. *Fam Med* 2016;**48**:775–83.

13 Wald HS, Borkan JM, Taylor JS, *et al.* Fostering and Evaluating Reflective Capacity in Medical Education: Developing the REFLECT Rubric for Assessing Reflective Writing. *Acad Med* 2012;**87**:41–50. doi:10.1097/ACM.0b013e31823b55fa

14 Uygur J, Stuart E, De Paor M, *et al.* A Best Evidence in Medical Education systematic review to determine the most effective teaching methods that develop reflection in medical students: BEME Guide No. 51. *Med Teach* 2019;**41**:3–16. doi:10.1080/0142159X.2018.1505037

15 Nicolaides M, Cardillo L, Theodoulou I, *et al.* Developing a novel framework for non-technical skills learning strategies for undergraduates: A systematic review. *Ann Med Surg* 2018;**36**:29–40. doi:10.1016/j.amsu.2018.10.005

16 Laidlaw A, Salisbury H, Doherty EM, *et al.* National survey of clinical communication assessment in medical education in the United Kingdom (UK). *BMC Med Educ* 2014;**14**:10. doi:10.1186/1472-6920-14-10

17 Martino S, Haeseler F, Belitsky R, *et al.* Teaching brief motivational interviewing to Year three medical students. *Med Educ* 2007;**41**:160–7. doi:10.1111/j.1365-2929.2006.02673.x

18 Locklear W, Meires J, Quallich SA. Letters to the Editor. *Urol Nurs* 2018;**38**:307. doi:10.7257/1053-816x.2018.38.6.307

19 Ayuob NN, Qadi MA, Eldeek BS, *et al.* Evaluation of a communication skills training course for medical students using peer role-play. *J Pak Med Assoc* 2017;**67**:745–51.

20 Roze Des Ordons AL, Doig CJ, Couillard P, *et al.* From Communication Skills to Skillful Communication: A Longitudinal Integrated Curriculum for Critical Care

For peer review only - http://bmjopen.bmj.com/site/about/guidelines.xhtml

Medicine Fellows. *Acad Med* 2017;**92**:501–5. doi:10.1097/ACM.0000000000001420

21 Simmenroth-Nayda A, Heinemann S, Nolte C, *et al.* Psychometric properties of the Calgary Cambridge guides to assess communication skills of undergraduate medical students. *Int J Med Educ* 2014;**5**:212–8. doi:10.5116/ijme.5454.c665

22 Kurtz SM, Silverman JD. The Calgary-Cambridge Referenced Observation Guides: an aid to defining the curriculum and organizing the teaching in communication training programmes. *Med Educ* 1996;**30**:83–9. doi:10.1111/j.1365-2923.1996.tb00724.x

23 Stewart M, Brown JB, Weston WW, *et al. Patient-Centered Medicine: Transforming the Clinical Method*. Third Edit. London: : Radcliffe Publishing 2014.

24 Franco C, Franco R, Severo M, *et al.* A Brief Course on Clinical Communication Skills: A Multi- Centered Study. *Acta Med Port* 2016;**29**:809–18. doi:10.20344/amp.8393

25 Meeus W, Van Petegem P, Engels N. Validity and reliability of portfolio assessment in pre-service teacher education. *Assess Eval High Educ* 2009;**34**:401–13. doi:10.1080/02602930802062659

26 Franco CAG dos S, Franco RS, Santos VM dos, *et al.* OSCE para Competências de Comunicação Clínica e Profissionalismo: Relato de Experiência e Meta-Avaliação. *Rev Bras Educ Med* 2015;**39**:433–41. doi:10.1590/1981-52712015v39n3e02832014

27 Makoul G. Essential elements of communication in medical encounters: the Kalamazoo consensus statement. *Acad Med* 2001;**76**:390–3.

28 Haggarty L. What is content analysis? *Med Teach* 1996;**18**:99–101. doi:10.3109/01421599609034141

29 Plack MM, Driscoll M, Marquez M, *et al.* Assessing Reflective Writing on a Pediatric Clerkship by Using a Modified Bloom's Taxonomy. *Ambul Pediatr* 2007;**7**:285–91. doi:10.1016/j.ambp.2007.04.006

30 Barton G, Ryan M. Multimodal approaches to reflective teaching and assessment in higher education. *High Educ Res Dev* 2014;**33**:409–24. doi:10.1080/07294360.2013.841650

31 Hulsman RL. Shifting goals in medical communication. Determinants of goal

For peer review only - http://bmjopen.bmj.com/site/about/guidelines.xhtml

detection and response formation. *Patient Educ Couns* 2009;**74**:302–8. doi:10.1016/j.pec.2008.12.001

32 Miller GE. The assessment of clinical skills/competence/performance. *Acad Med* 1990;**65**:S63-7.

33 Pangaro L, ten Cate O. Frameworks for learner assessment in medicine: AMEE Guide No. 78. *Med Teach* 2013;**35**:e1197–210. doi:10.3109/0142159X.2013.788789

34 Taylor DCMM, Hamdy H. Adult learning theories: implications for learning and teaching in medical education: AMEE Guide No. 83. *Med Teach* 2013;**35**:e1561-72. doi:10.3109/0142159X.2013.828153

35 Hayton A, Kang I, Wong R, *et al.* Teaching Medical Students to Reflect More Deeply. *Teach Learn Med* 2015;**27**:410–6. doi:10.1080/10401334.2015.1077124

36 Wear D, Zarconi J, Garden R, *et al.* Reflection in/and Writing: Pedagogy and Practice in Medical Education. *Acad Med* 2012;**87**:603–9. doi:10.1097/ACM.0b013e31824d22e9

37 Manca A, Gormley GJ, Johnston JL, *et al.* Honoring Medicine's Social Contract. *Acad Med* 2019;:1. doi:10.1097/ACM.0000000000003059

38 Wald HS. Refining a definition of reflection for the being as well as doing the work of a physician. *Med Teach* 2015;**37**:696–9. doi:10.3109/0142159X.2015.1029897

39 Mamede S, van Gog T, Moura AS, *et al.* Reflection as a strategy to foster medical students' acquisition of diagnostic competence. *Med Educ* 2012;**46**:464–72. doi:10.1111/j.1365-2923.2012.04217.x

40 Finlay IG, Maughan TS, Webster DJ. Portfolio learning: a proposal for undergraduate cancer teaching. *Med Educ* 1994;**28**:79–82. doi:10.1111/j.1365-2923.1994.tb02690.x

41 Cruess RL, Cruess SR, Steinert Y. Amending Miller's Pyramid to Include Professional Identity Formation. *Acad Med* 2015;**91**:180–5. doi:10.1097/ACM.0000000000000913

42 Al-Eraky M, Marei H. A fresh look at Miller's pyramid: assessment at the 'Is' and 'Do' levels. *Med Educ* 2016;**50**:1253–7. doi:10.1111/medu.13101

43 Lombarts MJMH. Competence-based education misses the essence of the medical profession. *Perspect Med Educ* 2015;**4**:326–8. doi:10.1007/s40037-015-0233-5

For peer review only - http://bmjopen.bmj.com/site/about/guidelines.xhtml

44 Charon R, Hermann N. Commentary: A Sense of Story, or Why Teach Reflective Writing? *Acad Med* 2012;**87**:5–7. doi:10.1097/ACM.0b013e31823a59c7

45 de la Croix A, Veen M. The reflective zombie: Problematizing the conceptual framework of reflection in medical education. *Perspect Med Educ* 2018;**7**:394–400. doi:10.1007/s40037-018-0479-9

46 Baernstein A, Fryer-Edwards K. Promoting reflection on professionalism: a comparison trial of educational interventions for medical students. *Acad Med* 2003;**78**:742–7.

47 Sandars J, Homer M. Reflective learning and the Net Generation. *Med Teach* 2008;**30**:877–9. doi:10.1080/01421590802263490

48 Tsingos-Lucas C, Bosnic-Anticevich S, Schneider CR, *et al.* Using Reflective Writing as a Predictor of Academic Success in Different Assessment Formats. *Am J Pharm Educ* 2017;**81**:8. doi:10.5688/ajpe8118

For peer review only

Table 1. Description of the five dimensions in the RS.

Dimensions	Descriptions of the Dimensions
1) Writing spectrum:	Assesses if reflection is just a superficial description of the event or a more detailed description with the exploitation of values and criticism.
2) Presence:	Assesses how much the student gets involved in or is detached from the narrative.
3) Description of conflict or disorienting dilemma:	Evaluates a wide range of situations, from the non-identification of a dilemma to a full description that includes different perspectives.
4) Attending to emotions:	Evaluates non-consideration of emotions, through the analysis of emotion.
5) Analysis and meaning making:	Evaluates the significance of the experience mentioned by the participant.
6) Attention to assignment-optional criterion:	Evaluates whether the student responds to what is sought.

For peer review only - http://bmjopen.bmj.com/site/about/guidelines.xhtml

Table 2. Example of fragments according to thematic categories.

Thematic Categories	Fragment Example
Non-Verbal	'I quickly noticed a strange, slightly frightened look on his face…'.
Steps of Consultation:	'…the consultation I performed was... like a questionnaire application...'.
Doctor-Patient Relationship:	'...it helps me, mainly to understand how to put the patient's needs and well-being above my own...'.
Empathy and Respect:	'I believe it is consensual that the attitude of the… …is subject to criticism, after all, respect and patience with the patient are prerequisites…'.
Humanistic Values:	'…the way he introduced himself... the attention with which he listened…'.

For peer review only

Table 3. Pearson correlations between the different methods of assessment.

**Assessment Methods**	OSCE	**p-Value**	MCQ	**p-Value**	REFLECT Score	**p-Value**
**MCQ**	0.396 (n=69)	**0.001***	-	**-**	-	**-**
**REFLECT Score**	0.250 (n=32)	**0.168**	-0.219 (n=32)	**0.228**	-	**-**
**Thematic Score**	0.412 (n=32)	**0.019***	0.439 (n=32)	**0.012***	0.410 (n=32)	**0.020***

MCQ; the p-value was considered a sign of statistical significance when it was lower than 0.05.

For peer review only

Reporting checklist for cohort study.

Based on the STROBE cohort guidelines.

Instructions to authors

Complete this checklist by entering the page numbers from your manuscript where readers will find each of the items listed below.

Your article may not currently address all the items on the checklist. Please modify your text to include the missing information. If you are certain that an item does not apply, please write "n/a" and provide a short explanation.

Upload your completed checklist as an extra file when you submit to a journal.

In your methods section, say that you used the STROBE cohortreporting guidelines, and cite them as:

von Elm E, Altman DG, Egger M, Pocock SJ, Gotzsche PC, Vandenbroucke JP. The Strengthening the Reporting of Observational Studies in Epidemiology (STROBE) Statement: guidelines for reporting observational studies.

		Reporting Item	Page Number
**Title and abstract**			
Title	#1a	Indicate the study’s design with a commonly used term in the title or the abstract	1
Abstract	#1b	Provide in the abstract an informative and balanced summary of what was done and what was found	4
**Introduction**			
Background / rationale	#2	Explain the scientific background and rationale for the investigation being reported	5
Objectives	#3	State specific objectives, including any prespecified hypotheses	7
**Methods**			
Study design	#4	Present key elements of study design early in the paper	8
Setting	#5	Describe the setting, locations, and relevant dates, including periods of recruitment, exposure, follow-up, and data collection	8

For peer review only

Eligibility criteria	#6a	Give the eligibility criteria, and the sources and methods of selection of participants. Describe methods of follow-up.	8
Eligibility criteria	#6b	For matched studies, give matching criteria and number of exposed and unexposed	8
Variables	#7	Clearly define all outcomes, exposures, predictors, potential confounders, and effect modifiers. Give diagnostic criteria, if applicable	9
Data sources / measurement	#8	For each variable of interest give sources of data and details of methods of assessment (measurement). Describe comparability of assessment methods if there is more than one group. Give information separately for for exposed and unexposed groups if applicable.	10
Bias	#9	Describe any efforts to address potential sources of bias	14
Study size	#10	Explain how the study size was arrived at	n/a - convenience sample
Quantitative variables	#11	Explain how quantitative variables were handled in the analyses. If applicable, describe which groupings were chosen, and why	8-10
Statistical methods	#12a	Describe all statistical methods, including those used to control for confounding	8-10
Statistical methods	#12b	Describe any methods used to examine subgroups and interactions	8-10
Statistical methods	#12c	Explain how missing data were addressed	8-10
Statistical methods	#12d	If applicable, explain how loss to follow-up was addressed	n/a
Statistical methods	#12e	Describe any sensitivity analyses	8-10
**Results**			
Participants	#13a	Report numbers of individuals at each stage of study—eg numbers potentially eligible, examined for eligibility, confirmed eligible, included in the study, completing follow-up, and analysed. Give	11

For peer review only

		information separately for for exposed and unexposed groups if applicable.	
Participants	#13b	Give reasons for non-participation at each stage	11
Participants	#13c	Consider use of a flow diagram	n/a - convenience sample
Descriptive data	#14a	Give characteristics of study participants (eg demographic, clinical, social) and information on exposures and potential confounders. Give information separately for exposed and unexposed groups if applicable.	11
Descriptive data	#14b	Indicate number of participants with missing data for each variable of interest	11
Descriptive data	#14c	Summarise follow-up time (eg, average and total amount)	11
Outcome data	#15	Report numbers of outcome events or summary measures over time. Give information separately for exposed and unexposed groups if applicable.	11-12
Main results	#16a	Give unadjusted estimates and, if applicable, confounder-adjusted estimates and their precision (eg, 95% confidence interval). Make clear which confounders were adjusted for and why they were included	11-12
Main results	#16b	Report category boundaries when continuous variables were categorized	11-12
Main results	#16c	If relevant, consider translating estimates of relative risk into absolute risk for a meaningful time period	11-12
Other analyses	#17	Report other analyses done—e.g., analyses of subgroups and interactions, and sensitivity analyses	11-12
**Discussion**			
Key results	#18	Summarise key results with reference to study objectives	12
Limitations	#19	Discuss limitations of the study, taking into account sources of potential bias or imprecision. Discuss both direction and magnitude of any potential bias.	14
Interpretation	#20	Give a cautious overall interpretation considering objectives,	12-13

For peer review only

		limitations, multiplicity of analyses, results from similar studies, and other relevant evidence.	
Generalisability	#21	Discuss the generalisability (external validity) of the study results	14
**Other Information**			
Funding	#22	Give the source of funding and the role of the funders for the present study and, if applicable, for the original study on which the present article is based	16

Notes:

- 10: n/a - convenience sample
- 13c: n/a - convenience sample The STROBE checklist is distributed under the terms of the Creative Commons Attribution License CC-BY. This checklist was completed on 27. March 2020 using https://www.goodreports.org/, a tool made by the EQUATOR Network in collaboration with Penelope.ai

For peer review only

BMJ Open

The added value of assessing medical students' reflective writings in communication skills training: a longitudinal study in four academic centres.

Journal:	*BMJ Open*
Manuscript ID	bmjopen-2020-038898.R1
Article Type:	Original research
Date Submitted by the Author:	13-Aug-2020
Complete List of Authors:	Ament Giuliani dos Santos Franco, Camila; Pontifical Catholic University of Parana, Medicine School<br>Franco, Renato; Pontifical Catholic University of Parana, Medicine School<br>Cecilio-Fernandes, Dario; State University of Campinas Faculty of Medical Sciences, ; University Of Groningen, University Medical Center Groningen<br>Severo, Milton; University of Porto Medical School, Department of Clinical Epidemiology, Predictive Medicine and Public Health and Public Health and Forensic Sciences, and Medical Education Department<br>Ferreira, Maria; University of Porto Faculty of Medicine, Public Health and Forensic Sciences, and Medical Education Department<br>de Carvalho-Filho, Marco Antonio; University of Minho School of Medicine, Internal Medicine; University Medical Center Groningen, CEDAR - Center for Educational Development and Research in Health Sciences
**Primary Subject Heading**:	Medical education and training
Secondary Subject Heading:	Communication
Keywords:	MEDICAL EDUCATION & TRAINING, MEDICAL ETHICS, PRIMARY CARE

SCHOLARONE™
Manuscripts

I, the Submitting Author has the right to grant and does grant on behalf of all authors of the Work (as defined in the below author licence), an exclusive licence and/or a non-exclusive licence for contributions from authors who are: i) UK Crown employees; ii) where BMJ has agreed a CC-BY licence shall apply, and/or iii) in accordance with the terms applicable for US Federal Government officers or employees acting as part of their official duties; on a worldwide, perpetual, irrevocable, royalty-free basis to BMJ Publishing Group Ltd ("BMJ") its licensees and where the relevant Journal is co-owned by BMJ to the co-owners of the Journal, to publish the Work in this journal and any other BMJ products and to exploit all rights, as set out in our licence.

The Submitting Author accepts and understands that any supply made under these terms is made by BMJ to the Submitting Author unless you are acting as an employee on behalf of your employer or a postgraduate student of an affiliated institution which is paying any applicable article publishing charge ("APC") for Open Access articles. Where the Submitting Author wishes to make the Work available on an Open Access basis (and intends to pay the relevant APC), the terms of reuse of such Open Access shall be governed by a Creative Commons licence – details of these licences and which Creative Commons licence will apply to this Work are set out in our licence referred to above.

Other than as permitted in any relevant BMJ Author's Self Archiving Policies, I confirm this Work has not been accepted for publication elsewhere, is not being considered for publication elsewhere and does not duplicate material already published. I confirm all authors consent to publication of this Work and authorise the granting of this licence.

For peer review only

The added value of assessing medical students' reflective writings in communication skills training: a longitudinal study in four academic centres.

Assessing reflective writings in communication skills training

Camila Ament Giuliani Franco

Medicine School, Pontifical Catholic University of Paraná, Curitiba, Brazil.

Address: Rua Imaculada Conceição, 1155 - Prado Velho, Curitiba – PR, 80215-901, Brasil - Escola de Medicina – PUCPR (Medicine School). Tel. +55 41 3271-1570. Email: camilaament@gmail.com; camila.giuliani@pucpr.br ORCID: https://orcid.org/0000-0003-1176-480X

Family Physician, MD, PhD. Professor at the School of Medicine (discipline of Family Medicine), Pontifical University of Paraná, Curitiba, Brazil. Public Health and Forensic Sciences, and Medical Education Department, University of Porto, Portugal.

The author declared no potential conflicts of interest with respect to the research, authorship, and/or publication of this article

Renato Soleiman Franco

Medicine School, Pontifical Catholic University of Paraná, Curitiba, Brazil.

Address: Rua Imaculada Conceição, 1155 - Prado Velho, Curitiba – PR, 80215-901, Brasil - Escola de Medicina – PUCPR (Medicine School). Tel. +55 41 3271-1570. Email: paum@uol.com.br; renato.soleiman@pucpr.br. ORCID: https://orcid.org/0000-0003-1176-480X

Psychiatrist, MD, PhD. Professor at the School of Medicine (discipline of Psychiatry), and at Post-Graduate Program in Bioethics - Pontifical University of Paraná, Curitiba, Brazil. Public Health and Forensic Sciences, and Medical Education Department, University of Porto, Portugal. Director of the Psychiatry Residency Program – FEAES/PMC, Curitiba.

For peer review only - http://bmjopen.bmj.com/site/about/guidelines.xhtml

The author declared no potential conflicts of interest with respect to the research, authorship, and/or publication of this article

Dario Cecilio-Fernandes

Department of Medical Psychology and Psychiatry, School of Medical Sciences, University of Campinas (UNICAMP), Campinas, SP, Brazil.

Address: R. Tessália Vieira de Camargo, 126 - Cidade Universitária, Campinas - SP, 13083-887. dario.fernandes@gmail.com. ORCID: https://orcid.org/0000-0002-8746-1680

Psychologist. PhD - Researcher at School of Medical Sciences, University of Campinas (UNICAMP), Campinas, SP, Brazil and Researcher, AFAMEE.

The author declared no potential conflicts of interest with respect to the research, authorship, and/or publication of this article

Milton Severo

Public Health and Forensic Sciences, and Medical Education Department. Faculty of Medicine, University of Porto, Portugal.

Address: Milton Severo, Departamento de Ciências da Saúde Pública e Forenses e Educação Médica, Piso 6, Faculdade de Medicina da Universidade do Porto, Alameda Prof. Hernâni Monteiro, 4200-319 Porto – Portugal. E-mail: severo.milton@gmail.com; milton@med.up.pt ORCID: https://orcid.org/0000-0002-5787-4871.

Graduated in Applied Mathematics and Computation, PhD in Public Health. Department of Epidemiology, Predictive Medicine and Public Health, Faculty of Medicine, University of Porto, Portugal. Public Health and Forensic Sciences, and Medical Education Department, University of Porto, Portugal. EPIUnit - Institute of Public Health, University of Porto, Portugal.

For peer review only - http://bmjopen.bmj.com/site/about/guidelines.xhtml

The author declared no potential conflicts of interest with respect to the research, authorship, and/or publication of this article.

Maria Amélia Ferreira

Public Health and Forensic Sciences, and Medical Education Department. Faculty of Medicine, University of Porto, Portugal.

Address: Departamento de Ciências da Saúde Pública e Forenses e Educação Médica, Piso 6, Faculdade de Medicina da Universidade do Porto, Alameda Prof. Hernâni Monteiro, 4200-319 Porto – Portugal. Email: mameliaferreira@gmail.com; mameliaferreira@med.up.pt. ORCID: https://orcid.org/0000-0001-6789-3796.

Physician, PhD in Medical Education. Public Health and Forensic Sciences, and Medical Education Department, University of Porto, Portugal.

The author declared no potential conflicts of interest with respect to the research, authorship, and/or publication of this article

Marco Antonio de Carvalho Filho (corresponding author)

University of Minho, Braga, Portugal and Center for Education Development and Research in Health Professions - Research Group LEARN - Lifelong Learning, Education & Assessment Research, University of Groningen, Groningen, The Netherlands.

Address: Antonius Deusinglaan, 4, 9713 AW, Groningen, The Netherlands. E-mail: m.a.de.carvalho.filho@umcg.nl. https://orcid.org/0000-0001-7008-4092

Emergency Physician. PhD, Associate Professor, Internal Medicine Department, Medical School, University of Minho, Portugal; and Senior Researcher, Center of Education Development and Research in Health Professions, Research Group LEARN, University Medical Center Groningen, The Netherlands.

For peer review only - http://bmjopen.bmj.com/site/about/guidelines.xhtml

The added value of assessing medical students' reflective writings in communication skills training: a longitudinal study in four academic centres.

Abstract

Objectives: This study describes the development and implementation of a model to assess students` communication skills highlighting the use of reflective writing. We aimed to evaluate the usefulness of the students' reflections in the assessment of communication skills.

Design: Third- and fourth-year medical students enrolled in an elective course on clinical communication skills development were assessed using different assessment methods.

Setting and Participants: The communication skills course was offered at four universities (three in Brazil and one in Portugal) and included 69 students.

Outcome measures: The students were assessed by a multiple-choice questionnaire (MCQ), an objective structured clinical examination (OSCE), and reflective writing narratives. The Cronbach's alpha, Dimensionality, and the Person's correlation were applied to evaluate the reliability of the assessment methods and their correlations. Reflective witting was assessed by applying the REFLECT Rubric (Reflect Score [RS]) to measure reflections' depth, and the Thematic Score (TS) to map and grade reflections` themes.

Results: The Cronbach alpha for the MCQ, OSCE global score, TS, and RS were respectively 0.697, 0,633, 0,784 and 0,850. The inter-observer correlation for the TS and RS were respectively 0,907 and 0,816. The assessment of reflection using the TS was significantly correlated with the MCQ (r=0.412; p=0.019), OSCE (0.439; p=0.012*), and RS (0.410; p=0.020). The RS did not correlate with the MCQ and OSCE.

Conclusions: Assessing reflection through mapping the themes and analysing the depth of reflective writing expands the assessment of communication skills. While the assessment of reflective themes is related to the cognitive and behavioural domains of learning, the reflective depth seems to be a specific competence, not correlated with other assessment methods - possibly a metacognitive domain.

Keywords: medical education & training; medical ethics; primary care.

For peer review only

Strengths and limitations of this study

- This study details the use of medical students’ reflective narratives in the assessment of communication skills.
- The assessment of the depth (profundity) and the themes (topics) of medical students' reflective narratives has an additional value compared to the traditional assessment methods used in communication skills training.
- The method utilized to assess the depth and themes of medical students’ reflective narratives showed good reliability.
- The participants were recruited from a convenience sample and further studies are needed to explore the added value of assessing medical students’ reflective narratives in a natural context.

1. Introduction

Clinical communication is essential for medical students and must extend well beyond the reproduction of behaviours and skills [1]. Competent doctors must adapt their communication to the specific needs of their patients [2]. In this regard, for medical students to become competent communicators, they must reflect on their experiences with patients aiming for the self-monitoring of their thoughts and behaviours to improve their performance in further interactions with patients [1,3]. Although reflection is an essential component of developing communication [4], most communication skills training does not include the assessment of students’ reflections in their repertoire of assessment tools [5]. Understanding how assessing reflection may support (or not) the development of communication skills in medical students may offer medical educators a new strategy for improving doctor-patient communication.

Medical students must be aware of patients’ needs and willing to adapt their patterns of behaviour according these needs and context [6]. Although the learning of some basic behavioural rules can indeed be an excellent starting point, such rules governing behaviour may not suffice for guiding students in the process of navigating the complexity of doctor-

For peer review only - http://bmjopen.bmj.com/site/about/guidelines.xhtml

patient communication. Each patient is unique and has his or her system of beliefs and singular expectations. Doctors must tailor their communication strategies to match each patient needs while respecting his or her personality and social and cultural background [7,8]. Doctors should adapt their communication styles to each patient by addressing the complexity of human interactions, which includes attending with and regulating their own emotions, understanding the context, and identifying potential dilemmas. In mastering communication, doctors should reflect before, during and after each clinical encounter to recognise their limitations and identify areas for improvement while planning how to achieve better outcomes [4]. Therefore, we advocate that educational activities that target the development of medical students' communication skills should include the teaching and assessment of reflection.

Within an educational context, reflection is a process [9] whereby individuals critically analyse their cognitive and behavioural responses to a certain experience and develop a deeper understanding of the experience and themselves. The reflection may start even before the experience starts (*reflection-for-action*), so that students can achieve a broader understanding of a particular task, which helps them to prepare for action. For example, when students anticipate that the task exceeds their level of competence, they may ask for help [10]. The reflection can also occur during the experience (*reflection-in-action*). This reflection in action refers to the capacity to address just-in-time information by applying the process of analysis and critics during an event, which may lead to real-time adaptation of the performance. After the end of the experience, students can engage in a *reflection-on-action* process by reviewing and analysing the event and its course to reach a deeper understanding and elaborate new knowledge [8]. Fostering reflection on-action has been one of the starting points for the development of reflective practices in medical education, from first-year undergraduate classes to post-graduate training [3,11]. For instance, in the context of doctor-patient relationship, the process of reflection on-action has a vital role in building mental models that become available to be applied in future clinical experiences to enhance emotional awareness, emotion expression, and empathy [4,12–14].

Most of the methods for assessing reflection targets reflection on-action processes, mainly by the use of students' reflective writing [3,11]. Reflective writing supports students' self-monitoring, generates self-awareness [15] and promotes a deeper understanding of patients by allowing the inclusion of biopsychosocial perspectives in next consultations [16,17]. Although reflection on-action has been considered keen in the development of

For peer review only - http://bmjopen.bmj.com/site/about/guidelines.xhtml

clinical communication [4,18], its implementation has a low degree of systematisation and minimal attention has been paid to descriptions of the use of reflective writing as an assessment tool in this context [4].

Reflective writing can be assessed based on the content or depth of reflection. The content of reflection may be evaluated by theme or category-based analysis [19–21]. For example, Karnieli-Miller et al. (2018) used reflective writing to support the teaching of how to deliver bad news. In the reflective narratives, the authors identified through theme-based analysis all the elements that were part of the clinical protocol used as a reference during the study [20]. However, the study focused on the content of reflection, but not on the depth of reflection. Moreover, the authors did not compare the results of the assessment of the reflection with those obtained through other methods of assessment. Similar to Karnieli-Miller et al. (2018), Braverman et al. (2016) used a coded framework for the thematic analysis of third-year medical students' reflective writing on challenges in communicating with patients but also did not assess the depth of reflection [21]. Thus, the studies that have sought to determine the role of reflection in teaching communication have targeted its themes, rather than its depth.

The Reflection Evaluation for Enhanced Competencies Tool (REFLECT rubric), proposed by Wald et al. (2012), highlights the importance of deep reflection in the development of metacognition and effective patient care [22] and has been widely used to evaluate reflection, particularly reflection on-action processes [23]. These authors organised a multidimensional analysis of reflection that assesses five mandatory items: writing spectrum, presence, description of conflict, attending to emotions, and meaning-making [22]. These five items can be classified using a Likert scale ranging from 0 to 3, according to four different reflection levels (from non-reflective to critically reflective), which correspond to the depth of reflection. This assessment model distinguishes between written texts with only superficial reflection (descriptive) and those with a high density of reflective elements. Although the REFLECT rubric was used successfully in assessment strategies for different learning activities involving reflective writing in medical education, its use in communication skills training must be stimulated and better analysed [4,11].

Communication training traditionally applies a combination of multiple choice questionnaires (MCQs) and Objective Structured Clinical Examination (OSCE) stations to assess students' cognitive knowledge and check students' performance [24,25]. Previous

For peer review only - http://bmjopen.bmj.com/site/about/guidelines.xhtml

research shows a low correlation between the MCQ and OSCE scores, which suggests that, indeed, these methods are assessing different competencies [26–28]. Communication teachers should take advantage of these different scores and provide specific feedback targeting knowledge and/or behaviour. Since cultivating reflection skills is also relevant to the process of becoming a competent communicator, communication trainers should implement assessment strategies that target reflection skills to create an opportunity to provide feedback on this competency [29].

There is a lack of research exploring the impact of reflection on the learning of communication skills. The use of the reflective capacity in the teaching and assessment of communication skills, namely, in scenarios related to practice, must be encouraged once it can foster students' professionalism, critical thinking and attitudes [3,4,12–14,23]. Reflective capacity, as a metacognitive process, surpasses (but includes) cognitive and behavioural elements. Understanding the level of correlation between the scores for reflection and the scores for traditional assessments, such as MCQs and OSCEs, potentially contribute to the discussion regarding the role of assessing reflection in communication skills training. Therefore, we raise the following research questions: Is the assessment of reflective writing correlated with cognitive (MCQ) and behavioural (OSCE) assessment methods?

To address these questions, we report the development of a model for assessing the reflection on-action of medical students in the context of communication skills training by applying two methods to evaluate students' reflective writing (themes and depth). We also compare the assessment of reflective writing with other traditional methods (i.e., MCQ and OSCE) to understand the added value of assessing the reflection process using these two methods. Understanding the added value of assessing students' reflective writing may contribute to clarify the importance of reflection in the process of honing communication skills to improve doctor-patient communication and support its future application in learning activities.

For peer review only

2. Methods

2.1 Overview

This longitudinal observational study was carried out at three different Brazilian universities (one course at each university in 2015) and one university in Portugal (one course in 2016). Data collection occurred during these elective courses in clinical communication. Each course comprised five modules (25 hours in total) conducted over two months. The elective discipline did not disturb students' academic trajectory and occurred in parallel to the regular learning activities. It is worth mentioning that, although this course did not involve practice with patients, all of the students had clinical encounters with patients in hospitals and primary care settings during their regular academic activities.

The Calgary-Cambridge Guide to Communication [30,31] and Patient-Centred Medicine [32] were the conceptual and theoretical models behind this elective communication skills' course. The contents of the first four modules comprised the steps of consultation: 1) initiating the session, 2) gathering information, 3) explanation and planning, 4) closing the session; and last one included the 5) breaking bad news. These contents and models were employed as supportive frameworks, and students were not encouraged to follow them as behavioural protocols. The main focus of the course was on the need to reflect and adapt communication strategies to patients' needs and students' communication style. Each module of the course was structured following 4 steps: 1) presentation of the content via reflective, small-group discussions, 2) simulation activities with simulated patients; 3) Reflective debriefing and 4) Summary of the learning points and preparation for next-modules [33]. The course did not have a module about theoretical assumptions of reflection or reflective writing, but the instructor of the course structured the discussion of the content (step 1) and debriefing (step 3) using the Gibbs Reflective Circle [33].

The cases selected for simulation involved clinical scenarios about common health problems with contextual or emotional challenges. For example, in one scenario, an apparently healthy woman asked for a preconception consultation regarding planning for pregnancy. The woman had a history of sexual abuse (between the ages of eleven and thirteen) by her uncle. She was neglected by her family even after informing her parents about the abuse. This scenario is very emotional and, unfortunately, represents a common occurrence in primary care settings where the students have their clinical training. The learning objective of this scenario is to consider the patient as a whole (one of the main

For peer review only - http://bmjopen.bmj.com/site/about/guidelines.xhtml

principles of patient-centeredness), obtain biopsychosocial information and address emotions (discuss empathy and affective reactions). In preparation to engage with the scenarios, students are stimulated to reflect in action and develop self-awareness and active listening skills, both competencies are among the pillars of one of the theoretical references of the course. During the debriefing of this and other cases, the facilitator stimulated a profound, horizontal, and collaborative discussion about the different elements and emotions involved in dealing with the simulated encounter. The facilitator actively invited students to take different perspectives. Every session ended with the elaboration of an action plan aiming to improve student's future performance and provide better patient care. A detailed discussion of the course has been previously published [34].

2.2 Participants

A convenience sample of third- and fourth-year medical students at four universities were invited to participate in the study by email. For the sample recruitment, a class representative of the students in the third or fourth year sent an email to their colleagues inviting them to participate in the course. No financial incentives were given for their participation. A total of 69 participants (20 at University 1 - Brazil, 12 at University 2 - Brazil, 30 at University 3 - Brazil, and seven at University 4 - Portugal) agreed to participate. The participants joined a course containing five encounters with a total of 25 hours. The 69 participants were assessed at the end of the course with an MCQ and OSCE on communication skills. The participants were invited (but not obligated) to write a reflective piece, and 37 students produced texts.

2.3 Material: Assessment Instruments

We compared three different assessment methods: a cognitive test based on an MCQ, an examination of communication skills based on the OSCE, and an assessment of reflection through reflective writing. The MCQ and OSCE were administered after the last meeting of the course on communication skills. The reflective writing was optional and could be undertaken by the students at any point during the communication skills' course. We decided

For peer review only

that the reflective writing would be optional to understand the students' disposition to engage with this assessment method [35].

The MCQ consisted of 63 items about clinical communication. The items were based on clinical situations or conceptual issues that were grounded in the Calgary-Cambridge Guide to Communication [30,31], Patient-Centred Medicine [32], and Kalamazoo Consensus [36].

The OSCE included six stations specifically designed to assess communication skills. The OSCE was based on the same references of the MCQ (Calgary-Cambridge Guide to Communication [30,31], Patient-Centred Medicine [32] and Kalamazoo Consensus [36]). Four of these stations had been tested by the authors in a pilot project [37]). To elaborate the six stations, two medical educators with expertise in OSCE and clinical communication collaborated to develop the stations and checklists. The OSCE targets behavioural domains (communication skills) and affective domains (empathy and compassion) both in the context of doctor-patient interactions. According to the blueprint based on the content of the course, the stations assessed students in scenarios in which they must break bad news to a patient's family, break bad news to a patient, gather information to reach a clinical diagnosis, engage in shared decision-making, address moral conflicts, and care for a patient with multiple complaints. There was one observer for each OSCE station who was responsible for filling out the assessment checklist. These checklists consisted of between six and 14 items depending on the station. Each item on the checklist was then classified on a Likert scale ranging from 0 to 2 points. The final score of each station was obtained by the mean of its items. The OSCE global score was calculated as a mean considering the six stations.

For the reflective writing component, students could choose any aspect of doctor-patient communication that they considered challenging in their clinical practice. The only advice was that students should find a calm place to write – a place that enables them to focus their attention on their writing with as few distractions as possible. Medical students did not take a course on reflection and reflective writing before this study. The students received the following instruction: 'Suggestion for reflection: 1) Describe the situation; 2) Point out the dilemmas, doubts, and questions raised; 3) Point out feelings and observations; 4) Analyse the situation from different points of view; 5) Make a conclusion; and 6) Suggest a hypothesis. These steps are only a suggestion; you may conduct the reflection in whichever way that you prefer'.

For peer review only - http://bmjopen.bmj.com/site/about/guidelines.xhtml

The writing content was related to communication skills and evaluated (1) through the sum of the themes covered in each one of reflections – the thematic score (TS), and (2) through the REFLECT Rubric – the reflect score (RS) [22]. In the next paragraphs we describe how these two scores were calculated.

For establishing the TS, two researchers (CF and RF) started a content analysis individually by reading carefully all the reflective writings made by the students. After reading, CF and RF selected the fragments related to clinical communication [38] and generated a single list with all the fragments from the reflections of all students. Next, CF and RF grouped the fragments in thematic categories independently. After, CF and RF met to reach a consensus on the main themes. After the definition of the main thematic categories, CF and RF read each one of the reflective writings for a second time and decided whether each of the themes were present or not. The two researchers assigned point scores accordingly to the presence of a certain theme ('0' for absent and '1' for present). The final TS corresponded to the sum of all the themes approached by the student. Finally, the agreement between the two researchers was evaluated, and, when there was a difference between the two, a final TS was reached by consensus.

The assessment based on the five mandatory dimensions of the REFLECT Rubric followed the guidelines set by the authors of the rubric. The five mandatory dimensions are: description, presence, identification of a dilemma, emotion, and the meaning of the experience. Each one of the dimensions are evaluated considering four levels of reflective capacity scored from 0-3 (habitual action or nonreflective = 0, thoughtful action or introspection = 1, reflection = 2 and critical reflection = 3). The sum of the scores obtained in each dimension was the total RS. Appendix 1 presented a fragment of one reflective writing and the application of the assessment to the five dimensions of the REFLECT rubric (Appendix 1).

In summary, the TS refers to 'the subject of reflection – number of themes', the RS refers to 'how the reflection took place or the depth of reflection'.

For peer review only

2.4 Analysis

The quality of the MCQ was assessed by internal consistency, items' responsiveness, face, content, and construct validity. The face and content validity of MCQ were developed with the support of the group in the Medical Education Department of the University of Porto, which was responsible for the evaluation of high-stakes exams of the Faculty of Medicine to guarantee the quality of the items. Three experts in communication (one of them is an external member of the University) assessed and approved the assessment regarding its content. The internal consistency of the items was evaluated by Cronbach's alpha. The responsiveness and construct validity were evaluated according to a published study, in which this MCQ test was applied [34]. The items' responsiveness was considered adequate once the score before and after a course on communication improved significantly. The mean of improvement was 18.9% (confidence interval of 95%, ranges from 15.8 to 22.1%) ($p < 0.001$). The MCQ (pre and post-test) was applied to medical students who attained the same communication course at 4 universities. The improvement in the scores after the course did not show differences among universities ($p = 0.102$). Thus, the results indicate an acceptable construct validity.

The psychometric quality of the OSCE was evaluated by validation of the content (applying the principal component analysis for dimensionality) and internal consistency. Dimensionality was assessed using a scree plot, and the number of components was assessed according to the 'elbow rule'. An element or item was considered to contribute to a principal component when it had a correlation value higher than 0.30. Internal consistency was evaluated using Cronbach's alpha (Cronbach 1951). Acceptable values for internal consistency were considered to be higher than 0.7. The linear associations between the assessment methods were assessed using the Pearson's correlation considering missing complete at random to handle with missed correlations. It was also provided a 95% confidence interval for the Pearson's correlation to present the precision of the correlation.

To measure agreement between researchers, we used the intraclass single average value for absolute agreement. The inter-rater agreement rate was calculated for encoded fragments (TS) and for the RS. NVivo software (version 11.3.2 for Mac) was used for qualitative data analysis, while the SPSS, Version 25.0, was used for quantitative data analysis.

For peer review only - http://bmjopen.bmj.com/site/about/guidelines.xhtml

This research was approved by the Ethics Centre of the São João Hospital Centre of the Faculty of Medicine of the University of Porto (FMUP) and by the Research and Ethics Commission of the Pontifical Catholic University of Paraná (PUCPR). Participant consent was requested in the form of an informed consent before the participation in the communication skills course. Signed written consent forms were completed by all participants.

2.5 Patient and Public Involvement

There is no patient involved in the study

3. Results

Sixty-nine students followed the courses and were included in the study. Fifty-five of the students were women (79.7%), and the mean age of participants was 23.5 years (SD 2.495). Fourth-year students were the largest cohort (69.6%). All participants (69 students) underwent the MCQ and OSCE examinations, and 32 students also performed the reflective writing.

3.1 Quality of the Instruments

The MCQ examination had a Cronbach's alpha of 0.697. For the six OSCE stations, the lower Cronbach's alpha level was 0.702, and the higher was 0.815. The Cronbach's alpha of the OSCE global score was 0.633. Considering one component (OSCE global score), the factor loads of the OSCEs stations were higher than 0.3 (Table X).

The TS had a Cronbach's alpha of 0.784, while the inter-examiner correlation for absolute single-measure concordance was 0.907 (two examiners). The RS had a Cronbach's alpha of 0.850 and an inter-examiner correlation for absolute single-measure concordance of 0.816 (two examiners).

For peer review only - http://bmjopen.bmj.com/site/about/guidelines.xhtml

3.2 Thematic Analysis

The thematic categories of the reflections were non-verbal communication (NV), the patient's perspective (PP), steps of communication (SC), doctor-patient relationships (DPR), ethics and respect (ER), empathy and altruism (EA) and humanistic values (HV) (Table 1).

[Table 1 near here]

3.3 Correlation between Instruments

Table 2 shows the correlations between the four different assessment methodologies. There was no correlation between the score for the depth of reflection (RS) and both the MCQ and OSCE scores. The RS was only correlated with the thematic score (TS). However, the TS score was positively correlated with the MCQ score (0.439; p=0.012) and the OSCE score (0.412; p=0.019).

[Table 2 near here]

4. Discussion

The assessment of the depth and themes of reflection on-action provides a different perspective on the teaching and learning of communication skills. We found a positive correlation between the content of the students' reflections with their performance on a cognitive test and OSCE assessment, which suggested that the scope of the reflection was related to the students' knowledge. The lack of correlation between the depth of reflection and cognitive and behavioural tests suggests that reflection could be a particular competence domain.

4.1 Importance of Including Assessment of Depth and Content When Evaluating Reflection

For peer review only - http://bmjopen.bmj.com/site/about/guidelines.xhtml

For peer review only

The reflection process ranges from elementary cognitive levels (description, identification, knowledge, and others) to higher levels of processing, such as analysis, evaluation, synthesis, and creation [38]. Using different methods to assess reflection offers an effective strategy to encourage students to engage in reflective activities and enhances the probability of students reaching deeper levels of reflection [39]. Thus, we agree with Hulsman et al. (2009) and advocate for the assessment of reflection in terms of its depth and content (themes) to improve communication skills training [40].

In the assessment of the reflection themes, teachers map the topics students address in their reflections. We observed that the number of themes addressed by students are linked to both knowledge [38] and practical performance [41–43]. Our results suggest that a broader knowledge base and a bigger repertoire of adequate behaviours help students to respond appropriately to different practical situations. The analysis of reflections that are based on its themes can be applied to assist the evaluation of these learning elements.

Interestingly, the reflection depth seems to be a different competence, not necessarily related to the knowledge level or current performance, but possibly related to the values and attitudes of the student regarding a specific topic [40]. It is possible that assessing the depth of reflective writings, even in a particular context (communication skills in our case), enables the evaluation of a specific domain of competence (reflective competence or reflective capacity). Aligned with this hypothesis, Moniz et al. (2015) showed a lack of correlation between the depth of reflection (RS) and OSCE and MCQ scores of undergraduate medical students. However, in Moniz`s study, the assessment methods were not targeting the same competence and the absence of standardization could explain the observed lack of correlation. In our study, we assessed a singular set of competencies (communication skills) and observed the same lack of correlation between reflection depth and other assessment methods. Thus, even after narrowing the context, the lack of association persists.

Learning is a lifelong enterprise and achieving deeper reflection is crucial to the process of becoming an independent and self-regulated learner [44].The achievement of deeper reflection requires (1) understanding the context; (2) elaborating on the experience; (3) searching for solutions to the problems posed; (4) acknowledging the different subjects involved; and (5) taking different perspectives [45]. Thus, when doctors achieve a deep sense of reflection on their practice they move from a state of being knowledge consumers to become active professionals capable of transforming their reality aiming for a practice based

For peer review only - http://bmjopen.bmj.com/site/about/guidelines.xhtml

on their values and centred on the patient [46]. We believe that deeper reflection goes beyond applying the knowledge to a fixed situation; deeper reflection incorporates the elaboration of new knowledge, balance of different perspectives, anticipation of challenges and planning of future behaviour [47].

Adding the depth of reflection to teaching and assessment models may allow teachers to capture students' standpoint, their meaning-making processes, and their values [48]. We hypothesize that the depth of reflection, particularly concerning communication skills, could be linked to the domain of 'being a doctor' and the formation of professional identity [49,50] by involving elements that extend beyond the context of daily practice to include belief systems and values, which are not commonly assessed in knowledge tests and OSCEs.

4.2 The Risks of Assessing Reflection

The assessment of reflection introduces the risk of limiting the reflective practice For instance, in our study, the observed lack of correlation with cognitive and behavioural assessments may derive from the failing of reflective writing to comprise all of the complexity related to the doctor-patient communication. In practical settings, when caring for a patient, students' reflective practice involves gathering information; being empathetic and compassionate; becoming aware of the clinical, emotional, and social context; and identifying conflicts—all crucial elements of addressing patients' needs to guarantee a patient-centred attitude. As a result, reflection is a complex process that involves emotional, cognitive, and moral dimensions. Considering this complexity, we must ponder to what extent the writing reflections are capable of capturing all the elements of students' reflective processes. In addition, our grading system may have driven students to focus on some aspects of the communication process while disregarding other aspects. Grading reflections can pressure students in scoring. The prevalent culture based on targeting high scores may motivate students to "play the game" and perform tasks and adopt behaviours to fit the expectations of teachers without engaging in transformative learning [51]. Thus, the lack of a correlation between the reflective capacity and knowledge and behaviour and the limits of assessing reflection must be considered. This lack of correlation cannot be extrapolated to the reflective capacity, which is an important limitation of our study. Nevertheless, it is essential to continue investigating the role of reflection as an assessment method for exploring the potential of reflective practice in medical education [52].

For peer review only - http://bmjopen.bmj.com/site/about/guidelines.xhtml

The risk of adopting a reductionistic approach to reflective practices may be avoided by driving the reflective process beyond the achievement of satisfactory grades and performance towards the questioning of taken-for-granted assumptions. These questionings must include the examination of power relations and social and systemic structures. Thus, the reflective capacity should not only address students' knowledge but also foster students' ability to critically analyse what is assumed to be right or wrong [46,53–56].

4.3 Limitations

This study is one of the first studies to apply multiple methods of assessment, including the evaluation of reflection on-action; however, its limits must be considered. The sample of this study was small and convenient. Our small sample may have influenced both the qualitative analysis and quantitative analysis. It is possible that larger samples could increase the number of categories and subcategories in the thematic analysis. Moreover, the lower number of assessments using the REFLECT rubric (32) restricts the generalisation of the results. The small sample limits the application of more refined statistical methods, for example, adjusting the results for sample characteristics. As it was self-selected, the sample may represent more knowledgeable and motivated students, which may influence both the scores and percentage of students who engaged in the reflective writing (higher than 50%). The fact that the reflection was optional could have attracted students who were naturally reflective, which can also be a confounder. Our results must be confirmed by investigations using non-convenient samples and with a greater number of participants.

The clinical practice involves a complex setting where elements beyond reflective capacity can drive decisions and behaviour, for example, emotional regulation and interpersonal skills. Thus, reflection during an event (reflection in-action) would arguably be more correlated with students' cognitive and behavioural developments. Note that the lack of correlation among the assessment methods relates to reflection on-action and does not relate to reflection in general. To broaden the applicability of reflection as an assessment method, future studies also need to focus on assessing reflection in-action processes.

The assessments were reliable and consistent but limited in terms of reproducibility owing to the number of assessments made. Our method of assessing reflection (reflective writing) could be an element of bias since studies show different results when different reflection methods are used. For example, when reflecting in interviews, students may show levels of reflection that are different from those shown in reflective writing [57]. The current

For peer review only - http://bmjopen.bmj.com/site/about/guidelines.xhtml

generation of students has a range of preferences when it comes to learning and methods of expression, and many do not have strong skills in written expression [58]. Thus, reflective depth can indeed be associated with students' writing skills [59]. In this way, some authors suggest diversification of reflective registers using alternatives such as digital storytelling. Thus, the use of writing to assume the depth of reflection has an important bias to be considered. Drawing definite conclusions about students' reflectiveness from only one source of reflective material may be biased.

Few studies apply multiple methods to assess communication skills, mainly studies that evaluate reflection. Although the results of this research highlight the assessment of reflections and promote discussions on its use for communication skills training, our assumptions and the limitations of this research may be considered.

4.5 Practical Implications

Becoming a good communicator is one of the challenges posed to medical students. Communication training already embraces a body of cognitive knowledge that grounds learning activities. Communication training has also developed different strategies to nurture, check, and give feedback on the behaviours and attitudes of medical students during role-playing and simulated or real clinical encounters. However, becoming a good communicator is a life-long process, and, after leaving medical school, junior doctors have to take control of their learning process. Developing a reflective mindset that is capable of evaluating current behaviour – its roots, professional and personal consequences, and emotional impact – will allow junior doctors to transform their understandings and attitudes towards more patient-centred care. Reflection can facilitate this trajectory by supporting medical students during their first steps in becoming autonomous critical thinkers.

Conclusion

This study supports the use of reflective narratives as a complementary assessment method in the context of communication skills training. Assessing the depth of reflection offers a new perspective on students' development and allows the teacher to dive into students' understandings of the value of becoming a good communicator.

For peer review only - http://bmjopen.bmj.com/site/about/guidelines.xhtml

Acknowledgements

We would also like to thank students for their willingness to participate in the course and achieve the assessment methods.

Author Statement

Franco, CAGS and Franco, RS participated in the conception, design of the study, acquisition of data, analysis and interpretation of data, writing the final version of the manuscript. Cecilio-Fernades, D made substantial contributions in the interpretation of data and revising the paper critically for important improvement in whole manuscript. Carvalho-Filho MA made substantial contributions in the analysis, interpretation of data and in the writing of the final version of the manuscript. Severo, M and Ferreira, MA made substantial contributions in the conception and design of the study, analysis and interpretation of data. All authors read and approved the final manuscript.

Declaration of Conflicting Interests

The author(s) declared no potential conflicts of interest with respect to the research, authorship, and/or publication of this article.

Funding

Financial support for the authors was provided by scholarships from the Conselho Nacional de Desenvolvimento Científico e Tecnológico (Brazilian National Council of Technological and Scientific Development - 229753/2013-2) and the Coordenação de Aperfeiçoamento de Pessoal de Nível Superior (Coordination for the Improvement of Higher Education Personnel, Brazil -13271/13-0).

Data availability statement: The datasets used during are available from the corresponding author on reasonable request.

Word Count: 3622 words

For peer review only

References

1 Stewart M, Brown JB, Donner A, *et al.* The impact of patient-centered care on outcomes. *J Fam Pract* 2000;**49**:796–804.http://www.ncbi.nlm.nih.gov/pubmed/11032203

2 Hoffmann TC, Bennett S, Tomsett C, *et al.* Brief training of student clinicians in shared decision making: a single-blind randomized controlled trial. *J Gen Intern Med* 2014;**29**:844–9. doi:10.1007/s11606-014-2765-5

3 Sandars J. The use of reflection in medical education: AMEE Guide No. 44. *Med Teach* 2009;**31**:685–95. doi:10.1080/01421590903050374

4 Karnieli-Miller O, Michael K, Gothelf AB, *et al.* The Associations between Reflective Ability and Communication Skills among Medical Students. *Patient Educ Couns* Published Online First: 2020. doi:10.1016/j.pec.2020.06.028

5 Carney PA, Palmer RT, Fuqua Miller M, *et al.* Tools to Assess Behavioral and Social Science Competencies in Medical Education. *Acad Med* 2016;**91**:730–42. doi:10.1097/ACM.0000000000001090

6 Deveugele M. Communication training: Skills and beyond. *Patient Educ Couns* 2015;**98**:1287–91. doi:10.1016/j.pec.2015.08.011

7 Sommer J, Lanier C, Perron NJ, *et al.* A teaching skills assessment tool inspired by the Calgary-Cambridge model and the patient-centered approach. *Patient Educ Couns* 2016;**99**:600–9. doi:10.1016/j.pec.2015.11.024

8 Mamede S, Schmidt HG. The structure of reflective practice in medicine. *Med Educ* 2004;**38**:1302–8. doi:10.1111/j.1365-2929.2004.01917.x

9 Nguyen QD, Fernandez N, Karsenti T, *et al.* What is reflection? A conceptual analysis of major definitions and a proposal of a five-component model. *Med Educ* 2014;**48**:1176–89. doi:10.1111/medu.12583

10 Schön DA. *The reflective practitioner: How professionals think in action*. 1985. doi:10.1109/PROC.1985.13210

11 Karnieli-Miller O. Reflective practice in the teaching of communication skills. *Patient*

For peer review only

Educ Couns Published Online First: July 2020. doi:10.1016/j.pec.2020.06.021

12 Shapiro J, Kasman D, Shafer A. Words and Wards: A Model of Reflective Writing and Its Uses in Medical Education. *J Med Humanit* 2006;**27**:231–44. doi:10.1007/s10912-006-9020-y

13 Walling A, Shapiro J, Ast T. What makes a good reflective paper? *Fam Med* 2013;**45**:7–12.

14 Chen I, Forbes C. Reflective writing and its impact on empathy in medical education: systematic review. *J Educ Eval Health Prof* 2014;**11**:20. doi:10.3352/jeehp.2014.11.20

15 Embo MPC, Driessen E, Valcke M, *et al.* Scaffolding reflective learning in clinical practice: A comparison of two types of reflective activities. *Med Teach* 2014;**36**:602–7. doi:10.3109/0142159X.2014.899686

16 Caeiro C, Cruz EB, Pereira CM. Arts, literature and reflective writing as educational strategies to promote narrative reasoning capabilities among physiotherapy students. *Physiother Theory Pract* 2014;**30**:572–80. doi:10.3109/09593985.2014.928919

17 Muneeb A. The Art of Healing through Narrative Medicine in Clinical Practice: A Reflection. *Perm J* Published Online First: 2017. doi:10.7812/TPP/17-013

18 Larsen DP, London DA, Emke AR. Using reflection to influence practice: student perceptions of daily reflection in clinical education. *Perspect Med Educ* 2016;**5**:285–91. doi:10.1007/s40037-016-0293-1

19 Karnieli-Miller O, Palombo M, Meitar D. See, reflect, learn more: qualitative analysis of breaking bad news reflective narratives. *Med Educ* 2018;**52**:497–512.http://doi.wiley.com/10.1111/medu.13582

20 Karnieli-Miller O, Michael K, Eidelman S, *et al.* What you "see" is how you communicate: Medical students' meaning making of a patient's vignette. *Patient Educ Couns* 2018;**101**:1645–53. doi:10.1016/j.pec.2018.04.004

21 Braverman G, Bereknyei Merrell S, Bruce JS, *et al.* Finding the Words: Medical Students' Reflections on Communication Challenges in Clinic. *Fam Med* 2016;**48**:775–83.https://www.cochranelibrary.com/central/doi/10.1002/central/CN-01606071/full

For peer review only - http://bmjopen.bmj.com/site/about/guidelines.xhtml

22 Wald HS, Borkan JM, Taylor JS, *et al.* Fostering and Evaluating Reflective Capacity in Medical Education: Developing the REFLECT Rubric for Assessing Reflective Writing. *Acad Med* 2012;**87**:41–50. doi:10.1097/ACM.0b013e31823b55fa

23 Uygur J, Stuart E, De Paor M, *et al.* A Best Evidence in Medical Education systematic review to determine the most effective teaching methods that develop reflection in medical students: BEME Guide No. 51. *Med Teach* 2019;**41**:3–16. doi:10.1080/0142159X.2018.1505037

24 Nicolaides M, Cardillo L, Theodoulou I, *et al.* Developing a novel framework for non-technical skills learning strategies for undergraduates: A systematic review. *Ann Med Surg* 2018;**36**:29–40. doi:10.1016/j.amsu.2018.10.005

25 Laidlaw A, Salisbury H, Doherty EM, *et al.* National survey of clinical communication assessment in medical education in the United Kingdom (UK). *BMC Med Educ* 2014;**14**:10. doi:10.1186/1472-6920-14-10

26 Martino S, Haeseler F, Belitsky R, *et al.* Teaching brief motivational interviewing to Year three medical students. *Med Educ* 2007;**41**:160–7. doi:10.1111/j.1365-2929.2006.02673.x

27 Locklear W, Meires J, Quallich SA. Letters to the Editor. *Urol Nurs* 2018;**38**:307. doi:10.7257/1053-816x.2018.38.6.307

28 Ayuob NN, Qadi MA, Eldeek BS, *et al.* Evaluation of a communication skills training course for medical students using peer role-play. *J Pak Med Assoc* 2017;**67**:745–51.

29 Roze Des Ordons AL, Doig CJ, Couillard P, *et al.* From Communication Skills to Skillful Communication: A Longitudinal Integrated Curriculum for Critical Care Medicine Fellows. *Acad Med* 2017;**92**:501–5. doi:10.1097/ACM.0000000000001420

30 Simmenroth-Nayda A, Heinemann S, Nolte C, *et al.* Psychometric properties of the Calgary Cambridge guides to assess communication skills of undergraduate medical students. *Int J Med Educ* 2014;**5**:212–8. doi:10.5116/ijme.5454.c665

31 Kurtz SM, Silverman JD. The Calgary-Cambridge Referenced Observation Guides: an aid to defining the curriculum and organizing the teaching in communication training programmes. *Med Educ* 1996;**30**:83–9. doi:10.1111/j.1365-2923.1996.tb00724.x

32 Stewart M, Brown JB, Weston WW, *et al.* *Patient-Centered Medicine: Transforming*

For peer review only - http://bmjopen.bmj.com/site/about/guidelines.xhtml

the Clinical Method. Third Edit. London: : Radcliffe Publishing 2014.

33 Gibbs G. *Learning by doing: A guide to teaching and learning methods*. London, England, England: : FEU 1988.

34 Franco C, Franco R, Severo M, *et al.* A Brief Course on Clinical Communication Skills: A Multi- Centered Study. *Acta Med Port* 2016;**29**:809–18. doi:10.20344/amp.8393

35 Meeus W, Van Petegem P, Engels N. Validity and reliability of portfolio assessment in pre-service teacher education. *Assess Eval High Educ* 2009;**34**:401–13. doi:10.1080/02602930802062659

36 Makoul G. Essential elements of communication in medical encounters: the Kalamazoo consensus statement. *Acad Med* 2001;**76**:390–3.http://www.ncbi.nlm.nih.gov/pubmed/11299158

37 Franco CAG dos S, Franco RS, Santos VM dos, *et al.* OSCE para Competências de Comunicação Clínica e Profissionalismo: Relato de Experiência e Meta-Avaliação. *Rev Bras Educ Med* 2015;**39**:433–41. doi:10.1590/1981-52712015v39n3e02832014

38 Plack MM, Driscoll M, Marquez M, *et al.* Assessing Reflective Writing on a Pediatric Clerkship by Using a Modified Bloom's Taxonomy. *Ambul Pediatr* 2007;**7**:285–91. doi:10.1016/j.ambp.2007.04.006

39 Barton G, Ryan M. Multimodal approaches to reflective teaching and assessment in higher education. *High Educ Res Dev* 2014;**33**:409–24. doi:10.1080/07294360.2013.841650

40 Hulsman RL. Shifting goals in medical communication. Determinants of goal detection and response formation. *Patient Educ Couns* 2009;**74**:302–8. doi:10.1016/j.pec.2008.12.001

41 Miller GE. The assessment of clinical skills/competence/performance. *Acad Med* 1990;**65**:S63-7.http://www.ncbi.nlm.nih.gov/pubmed/2400509

42 Pangaro L, ten Cate O. Frameworks for learner assessment in medicine: AMEE Guide No. 78. *Med Teach* 2013;**35**:e1197–210. doi:10.3109/0142159X.2013.788789

43 Taylor DCMM, Hamdy H. Adult learning theories: implications for learning and

For peer review only

For peer review only - http://bmjopen.bmj.com/site/about/guidelines.xhtml

teaching in medical education: AMEE Guide No. 83. *Med Teach* 2013;**35**:e1561-72. doi:10.3109/0142159X.2013.828153

44 Hayton A, Kang I, Wong R, *et al.* Teaching Medical Students to Reflect More Deeply. *Teach Learn Med* 2015;**27**:410–6. doi:10.1080/10401334.2015.1077124

45 Wear D, Zarconi J, Garden R, *et al.* Reflection in/and Writing: Pedagogy and Practice in Medical Education. *Acad Med* 2012;**87**:603–9. doi:10.1097/ACM.0b013e31824d22e9

46 Manca A, Gormley GJ, Johnston JL, *et al.* Honoring Medicine's Social Contract. *Acad Med* 2019;:1. doi:10.1097/ACM.0000000000003059

47 Mamede S, van Gog T, Moura AS, *et al.* Reflection as a strategy to foster medical students' acquisition of diagnostic competence. *Med Educ* 2012;**46**:464–72. doi:10.1111/j.1365-2923.2012.04217.x

48 Finlay IG, Maughan TS, Webster DJ. Portfolio learning: a proposal for undergraduate cancer teaching. *Med Educ* 1994;**28**:79–82. doi:10.1111/j.1365-2923.1994.tb02690.x

49 Cruess RL, Cruess SR, Steinert Y. Amending Miller's Pyramid to Include Professional Identity Formation. *Acad Med* 2015;**91**:180–5. doi:10.1097/ACM.0000000000000913

50 Al-Eraky M, Marei H. A fresh look at Miller's pyramid: assessment at the 'Is' and 'Do' levels. *Med Educ* 2016;**50**:1253–7. doi:10.1111/medu.13101

51 Lombarts MJMH. Competence-based education misses the essence of the medical profession. *Perspect Med Educ* 2015;**4**:326–8. doi:10.1007/s40037-015-0233-5

52 McLeod GA, Barr J, Welch A. Best Practice for Teaching and Learning Strategies to Facilitate Student Reflection in Pre-Registration Health Professional Education: An Integrative Review. *Creat Educ* 2015;**06**:440–54. doi:10.4236/ce.2015.64044

53 Murdoch-Eaton D, Sandars J. Reflection: moving from a mandatory ritual to meaningful professional development. *Arch Dis Child* 2014;**99**:279–83. doi:10.1136/archdischild-2013-303948

54 Mann K, Gordon J, MacLeod A. Reflection and reflective practice in health professions education: a systematic review. *Adv Heal Sci Educ* 2009;**14**:595–621.

55 Harrison C, Wass V. The challenge of changing to an assessment for learning culture.

For peer review only - http://bmjopen.bmj.com/site/about/guidelines.xhtml

Med Educ 2016;**50**:704–6. doi:10.1111/medu.13058

56 Ng SL, Kinsella EA, Friesen F, *et al.* Reclaiming a theoretical orientation to reflection in medical education research: a critical narrative review. *Med Educ* 2015;**49**:461–75. doi:10.1111/medu.12680

57 Baernstein A, Fryer-Edwards K. Promoting reflection on professionalism: a comparison trial of educational interventions for medical students. *Acad Med* 2003;**78**:742–7.http://ovidsp.ovid.com/ovidweb.cgi?T=JS&PAGE=reference&D=emed6&NEWS=N&AN=2003298275

58 Sandars J, Homer M. Reflective learning and the Net Generation. *Med Teach* 2008;**30**:877–9. doi:10.1080/01421590802263490

59 Tsingos-Lucas C, Bosnic-Anticevich S, Schneider CR, *et al.* Using Reflective Writing as a Predictor of Academic Success in Different Assessment Formats. *Am J Pharm Educ* 2017;**81**:8. doi:10.5688/ajpe8118

For peer review only

Table 1. Example of fragments according to thematic categories.

Thematic Categories	Fragment Example
Non-Verbal	'I quickly noticed a strange, slightly frightened look on his face…'.
Steps of Consultation:	'…the consultation I performed was... like a questionnaire application...'.
Doctor-Patient Relationship:	'...it helps me, mainly to understand how to put the patient's needs and well-being above my own...'.
Empathy and Respect:	'I believe it is consensual that the attitude of the… …is subject to criticism, after all, respect and patience with the patient are prerequisites…'.
Humanistic Values:	'…the way he introduced himself... the attention with which he listened…'.

For peer review only

For peer review only - http://bmjopen.bmj.com/site/about/guidelines.xhtml

Table 2. Pearson correlations between the different methods of assessment.

**Assessment Methods**	OSCE	CI 95%	**p-Value**	MCQ	CI 95%	**p-Value**	REFLECT Score	CI 95%	**p-Value**
**MCQ**	0.396 (n=69)	0.17 to 0.59	**0.001***	-		**-**	-		**-**
**REFLECT Score**	0.250 (n=32)	-0.11 to 0.55	**0.168**	-0.219 (n=32)	-0.53 to 0.14	**0.228**	-		**-**
**Thematic Score**	0.412 (n=32)	0.07 to 0.66	**0.019***	0.439 (n=32)	0.11 to 0.68	**0.012***	0.410 (n=32)	0.07 to 0.66	**0.020***

*the p-value was considered a sign of statistical significance when it was lower than 0.05. CI: Confidence Interval for Pearson's correlation.

For peer review only - http://bmjopen.bmj.com/site/about/guidelines.xhtml

Appendix 1 - Scoring Reflective Writing using REFLECT rubric:

Reflective Writing: *I felt uncomfortable and it was hard for me stay present in the consultation because of the way the professor informed the diagnosis and managed the patient. Assessing the situation according to what physicians must do, several skills were not fulfilled in the patient care process: attention to patient well-being, autonomy and responsibility to promote better health for patients. The gathering of history by students had no benefit to the patient and only served a didactic function. As the diagnosis is cancer, which is stigmatised and has a very high negative charge (senior physician had performed a prior consultation and obtained all necessary information), it might not be the best time for medical students "to practice" history-taking. After our history-taking, the senior physician discussed the therapy for cancer with students and asked another physician to participate. They discussed the prognosis for the patient, suggested a new protocol in the research phase and assumed results that should not happen. All of these events occurred in front of the patient and their family. Adequate communication is important to adapt communication to each patient. Information must be provided according to subjects' needs and their capacity to understand… "Why to discuss in that way? They discussed uncertain things and affirmed the prognosis and other indications without scientific confirmation. It is difficult to evaluate these complex issues as students due to the scarce theoretical foundation for communication in medical school. The process of assimilation and application of role models prevails if there is no other point of criticism…*

1- **Writing Spectrum – Level: Reflection** ("movement beyond reporting or descriptive writing to reflecting; i.e., attempting to understand, question, or analyse an event"[1]). The fragments disposed of reveal that students wrote beyond the descriptive level. However, they did not explore and criticise the values, believes or assumptions behind the observed behaviour. Thus, this reflection exceeds the descriptive level and achieves reflection but not a critical reflection – the higher level for writing spectrum: *"The gathering of history by students had no benefit for the patient, but only a didactic function."; "As the diagnosis is cancer, which is stigmatised and has a very high negative charge (senior physician had conducted a prior consultation and obtained all necessary information), it might not be the best time for medical students "to practice" history-taking."*

2- **Presence – Level: Reflection** ("sense of writer being largely present"[1]) – The students presented the situation including her/himself in the situation, described the situation according to her/his point of view, which enabled an understanding of the participation of the student in the consultation. However, more details are needed to bring the reader to the setting, as expected for the Critical Reflection Level.

3- **Description of conflict or disorienting dilemma – Level: Reflection** ("description of the disorienting dilemma, conflict, challenge, or issue of concern"[1]) – The description includes the disorienting dilemma but does not include a more profound understanding of the "conflict, challenge, or issue of concern that includes multiple perspectives…" as expected for the next level: "Critical Reflection". There are three main dilemmas: the need to adapt the communication to each patient, the negative role models and the responsibility to patient well-being. All these elements were clearly stated in the text but lacked the necessary detail for Critical Reflection.

4- **Attending to Emotions – Level: Thoughtful action** ("recognition but no exploration or attention to emotions"[1]) – The students described his/her feeling and the narrative transmits his/her difficulty in handling emotions during the situation. However, no exploration was required for the next level of writing (Reflection) and beyond the recognition and insight on emotions necessary in Critical Reflection.

5- **Analysis and Meaning Making – Level: Reflection** ("some analysis and meaning-making"[1]) - The student noticed problems regarding communication and physicians' attitude. The writing suggests that the students recognised and analysed the situation; however, it could be more comprehensive for achieving Critical Reflection – for example, why did this doctor behave in this manner? The following fragments present some analysis of the student: *"it might not be the best time for medical students "to practice" history-taking..."; "To communicate adequately is important to adapt communication to each patient, and the information must be provided according to subjects' needs and the capacity to understand…".*

1- REFLECT rubric statements from: Wald, H. S., Borkan, J. M., Taylor, J. S., Anthony, D., & Reis, S. P. (2012). Fostering and Evaluating Reflective Capacity in Medical Education: Developing the REFLECT Rubric for Assessing Reflective Writing. Academic Medicine, 87(1), 41–50. The text in Italic correspond to the student reflective writing. The text in bold correspond to the REFLECT rubric items.

For peer review only - http://bmjopen.bmj.com/site/about/guidelines.xhtml

Reporting checklist for cohort study.

Based on the STROBE cohort guidelines.

Instructions to authors

Complete this checklist by entering the page numbers from your manuscript where readers will find each of the items listed below.

Your article may not currently address all the items on the checklist. Please modify your text to include the missing information. If you are certain that an item does not apply, please write "n/a" and provide a short explanation.

Upload your completed checklist as an extra file when you submit to a journal.

In your methods section, say that you used the STROBE cohortreporting guidelines, and cite them as:

von Elm E, Altman DG, Egger M, Pocock SJ, Gotzsche PC, Vandenbroucke JP. The Strengthening the Reporting of Observational Studies in Epidemiology (STROBE) Statement: guidelines for reporting observational studies.

		Reporting Item	Page Number
**Title and abstract**			
Title	#1a	Indicate the study's design with a commonly used term in the title or the abstract	1
Abstract	#1b	Provide in the abstract an informative and balanced summary of what was done and what was found	4
**Introduction**			
Background / rationale	#2	Explain the scientific background and rationale for the investigation being reported	5
Objectives	#3	State specific objectives, including any prespecified hypotheses	7
**Methods**			
Study design	#4	Present key elements of study design early in the paper	9
Setting	#5	Describe the setting, locations, and relevant dates, including periods of recruitment, exposure, follow-up, and data collection	9

For peer review only

Eligibility criteria	#6a	Give the eligibility criteria, and the sources and methods of selection of participants. Describe methods of follow-up.	10
Eligibility criteria	#6b	For matched studies, give matching criteria and number of exposed and unexposed	10
Variables	#7	Clearly define all outcomes, exposures, predictors, potential confounders, and effect modifiers. Give diagnostic criteria, if applicable	10-11
Data sources / measurement	#8	For each variable of interest give sources of data and details of methods of assessment (measurement). Describe comparability of assessment methods if there is more than one group. Give information separately for for exposed and unexposed groups if applicable.	10-11
Bias	#9	Describe any efforts to address potential sources of bias	13
Study size	#10	Explain how the study size was arrived at	n/a - convenience sample
Quantitative variables	#11	Explain how quantitative variables were handled in the analyses. If applicable, describe which groupings were chosen, and why	9-13
Statistical methods	#12a	Describe all statistical methods, including those used to control for confounding	9-13
Statistical methods	#12b	Describe any methods used to examine subgroups and interactions	9-13
Statistical methods	#12c	Explain how missing data were addressed	9-13
Statistical methods	#12d	If applicable, explain how loss to follow-up was addressed	n/a
Statistical methods	#12e	Describe any sensitivity analyses	9-13
**Results**			
Participants	#13a	Report numbers of individuals at each stage of study—eg numbers potentially eligible, examined for eligibility, confirmed eligible, included in the study, completing follow-up, and	14

For peer review only

		analysed. Give information separately for for exposed and unexposed groups if applicable.	
Participants	#13b	Give reasons for non-participation at each stage	14
Participants	#13c	Consider use of a flow diagram	n/a - convenience sample
Descriptive data	#14a	Give characteristics of study participants (eg demographic, clinical, social) and information on exposures and potential confounders. Give information separately for exposed and unexposed groups if applicable.	14
Descriptive data	#14b	Indicate number of participants with missing data for each variable of interest	14
Descriptive data	#14c	Summarise follow-up time (eg, average and total amount)	14
Outcome data	#15	Report numbers of outcome events or summary measures over time. Give information separately for exposed and unexposed groups if applicable.	14-15
Main results	#16a	Give unadjusted estimates and, if applicable, confounder-adjusted estimates and their precision (eg, 95% confidence interval). Make clear which confounders were adjusted for and why they were included	14-15
Main results	#16b	Report category boundaries when continuous variables were categorized	14-15
Main results	#16c	If relevant, consider translating estimates of relative risk into absolute risk for a meaningful time period	11-12
Other analyses	#17	Report other analyses done—e.g., analyses of subgroups and interactions, and sensitivity analyses	14-15
**Discussion**			
Key results	#18	Summarise key results with reference to study objectives	15
Limitations	#19	Discuss limitations of the study, taking into account sources of potential bias or imprecision. Discuss both direction and magnitude of any potential bias.	17-18

For peer review only

Interpretation	#20	Give a cautious overall interpretation considering objectives, limitations, multiplicity of analyses, results from similar studies, and other relevant evidence.	15-17
Generalisability	#21	Discuss the generalisability (external validity) of the study results	17-18
**Other Information**			
Funding	#22	Give the source of funding and the role of the funders for the present study and, if applicable, for the original study on which the present article is based	20

Notes:

- 10: n/a - convenience sample
- 13c: n/a - convenience sample The STROBE checklist is distributed under the terms of the Creative Commons Attribution License CC-BY. This checklist was completed on 27. March 2020 using https://www.goodreports.org/, a tool made by the EQUATOR Network in collaboration with Penelope.ai

For peer review only
